# Mindfulness-Based Interventions and the Hypothalamic–Pituitary–Adrenal Axis: A Systematic Review

**DOI:** 10.3390/neurolint16060115

**Published:** 2024-11-20

**Authors:** Hernando Vargas-Uricoechea, Alejandro Castellanos-Pinedo, Karen Urrego-Noguera, Hernando D. Vargas-Sierra, María V. Pinzón-Fernández, Ernesto Barceló-Martínez, Andrés F. Ramírez-Giraldo

**Affiliations:** 1 Metabolic Diseases Study Group, Department of Internal Medicine, Universidad del Cauca, Carrera 6 Nº 13N-50, Popayán 190001, Colombia; karenurrego@unicauca.edu.co (K.U.-N.); hdvargas@unicauca.edu.co (H.D.V.-S.); mpinzon@unicauca.edu.co (M.V.P.-F.); 2 Faculty of Health, Universidad de la Costa, Barranquilla 080003, Colombia; erbarcelo@yahoo.com (E.B.-M.); aramirez27@cuc.edu.co (A.F.R.-G.); 3 Faculty of Medicine, Universidad del Sinú, Hospital San Jerónimo, Montería 230001, Colombia; acaspinedo@yahoo.es; 4 Health Research Group, Department of Internal Medicine, Universidad del Cauca, Popayán 190003, Colombia

**Keywords:** mindfulness, cortisol, adrenal, stress

## Abstract

Background: Numerous studies have evaluated the effect that mindfulness-based interventions (MBIs) have on multiple health outcomes. For its part, stress is a natural response to environmental disturbances and within the associated metabolic responses, alterations in cortisol levels and their measurement in different tissues are a way to determine the stress state of an individual. Therefore, it has been proposed that MBIs can modify cortisol levels. Methods and results: The objective of this systematic review was to analyze and summarize the different studies that have evaluated the effect of MBIs on cortisol levels. The following databases were consulted: MEDLINE, AMED, CINAHL, Web of Science, Science Direct, PsycINFO, SocINDEX, PubMed, the Cochrane Library and Scopus. The search terms “mindfulness”, “mindfulness-based interventions” and “cortisol” were used (and the search was limited to studies from January 1990 to May 2024). In order to reduce selection bias, each article was scrutinized using the JBI Critical Appraisal Checklist independently by two authors. We included those studies with specified intervention groups with at least one control group and excluded duplicate studies or those in which the intervention or control group was not adequately specified. Significant changes in cortisol following MBIs were found in 25 studies, while 10 found no changes. The small sample size, lack of randomization, blinding, and probable confounding and interaction variables stand out in these studies. Conclusion: MBIs have biological plausibility as a means of explaining a positive effect on cortisol levels; however, the weakness of the studies and the absence of robust designs makes it difficult to establish a causal association between both variables. Registration number: INPLASY2024110017.

## 1. Introduction

There has been widespread academic debate in recent decades with respect to developing a universal definition of “mindfulness” as a construct. Although these definitions have tried to reach a consensus on a broader and more specific concept, many of them are far from adequate in terms of the true meaning of mindfulness [1,2,3].

Among the countless existing definitions, there is one that is widely accepted (and from which the others are derived): Mindfulness is a kind of present-centered, nonjudgmental and non-conceptual consciousness, in which each thought, feeling, or sensation that arises in the attentional field is recognized and accepted for what it is. There are several types of mindfulness-based interventions (MBIs), with some overlap between them (in their applications, indications and practice techniques). The most frequently studied are focused attention meditation, open monitoring meditation, loving-kindness meditation and body scan meditation. Among these MBIs, mindfulness-based stress reduction (MBSR) and mindfulness-based cognitive therapy (MBCT) stand out [1,2,3,4,5].

Briefly, MBSR consists of an eight-week plan of weekly group classes lasting 2 to 2.5 h (with a trained teacher), daily (audio-guided) practice at home (approximately 45 min a day) and a day-long mindfulness-based retreat. Similarly, MBCT integrates MBSR with cognitive science and cognitive behavioral therapy (CBT) and consists of eight weekly sessions of approximately 2 h in duration (each session), during which various formal and informal meditation practices are carried out [2,6,7,8,9,10,11].

MBIs have been widely used in recent decades in different clinical and biological settings and different educational, labor and business settings, among others. Multiple studies and reviews have examined the possible benefits of MBIs for outcomes such as stress, coping with illness, mood, depression, anxiety, post-traumatic stress disorder, self-compassion, memory and attention. Likewise, other outcomes, such as blood pressure levels, the risk of cardiovascular diseases and metabolic control, have been found to be improved in individuals with diabetes mellitus (and other health conditions) who have undergone MBIs [12,13,14,15,16,17,18,19].

Several outcomes have been shown to be positively associated with MBSR, including the control of chronic pain in individuals with fibromyalgia, arthritis and low back pain, as well as several markers of inflammation. Other studies have shown that MBCT can relieve symptoms of depression, anxiety, or fatigue and improve metabolic control in individuals with diabetes mellitus [20,21,22,23,24,25,26].

Similarly, both MBSR and MBCT can improve quality of life and different psychological and physical symptoms across a wide range of chronic somatic conditions (for example, cancer, cardiovascular disease, arthritis and chronic stress) [27,28,29].

To ensure survival, human beings must continually adapt to their internal and external environment (homeostasis). The maintenance of homeostasis requires the participation of countless biochemical processes and physiological mediators, where hormones such as cortisol and catecholamines play a primary role. The combination of these hormones and the activation of the autonomic nervous system (ANS) and the central nervous system allow for better adaptation and responses to daily activities [24,30,31].

The release and interaction between these physiological mediators and some immunological and metabolic parameters (allostasis) have both a protective and adaptive component for the species, as long as they are activated or inactivated in a balanced manner in the presence of a challenge or stress environment. However, when this response persists over time, it can be detrimental to human health and well-being [32,33].

In this sense, when we are faced with a stressful situation, two systems are physiologically activated: the sympathetic–adrenal–medullary system (SAM) and the hypothalamic–pituitary–adrenal (HPA) axis. Thus, cortisol has been associated with latent or chronic processes of stress, and it has been proposed that dysregulation of the HPA axis (related to deficiency or excess cortisol secretion) at least partly explains this association [34,35,36,37,38].

However, there is currently no gold standard biomarker available for assessing chronic stress (given its complex etiology and the individual variability in its signs and symptoms, which has been a real challenge for clinicians); however, it has been proposed that cortisol levels (based on the norm of having an intact HPA axis) reflect a sustained and prolonged (chronic) response to stress. Therefore, according to the relevance of the HPA axis in the response to chronic stress, it has been hypothesized that cortisol levels may be a useful marker in the evaluation of individuals with different degrees of stress [30,39,40].

The aims of this review were to analyze and summarize the different studies that have evaluated the effect of MBIs on cortisol levels and to elucidate the possible underlying biological mechanisms.

## 2. Methods and Results

We registered this systematic review with the “International Platform of Registered Systematic Review and Meta-Analysis Protocols INPLASY” (registration number: INPLASY2024110017. DOI number: 10.37766/inplasy2024.11.0017) and followed the “Reporting Checklist for Systematic Review. Based on the PRISMA guidelines” (Table S1).

A comprehensive and sensitive search for related evidence was performed in the following databases: MEDLINE, AMED, CINAHL, Web of Science, Science Direct, PsycINFO, SocINDEX, PubMed, the Cochrane Library and Scopus. The search terms “mindfulness”, “mindfulness-based interventions” and “cortisol” were used, and the search was limited to studies from January 1990 to May 2024.

Intervention studies with MBIs (ideally with control groups) were considered to be eligible; systematic reviews and meta-analyses were also reviewed and analyzed, and studies that could be useful in accordance with the requirements were extracted. Textbooks were also manually searched for information related to mindfulness, meditation and stress management, to deepen and expand the available evidence. We excluded duplicate studies or those in which the intervention or control group was not adequately specified.

In order to reduce selection bias, each article was scrutinized according to the JBI Critical Appraisal Checklist. The identification and selection of studies were carried out by the lead author (H. V-U) and HD. V-S. In cases where discrepancies arose in the extracted data, they collaboratively conducted a second round of extraction to validate the accuracy of the information.

**Figure 1:**
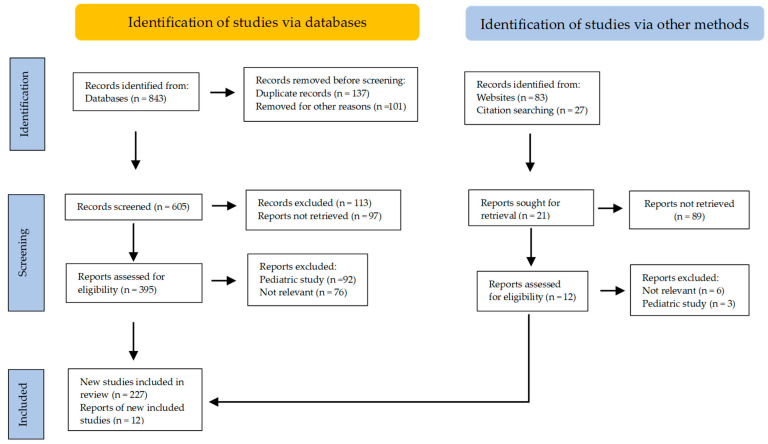
PRISMA flow diagram. Method for the selection of articles.

The following data were retrieved: authors, year of publication, subjects (age, gender, type of population), number of participants, study design, type of intervention, assessment of cortisol levels and results. Only English-language articles were included (Figure 1).

Data extraction was performed using a predefined data form created in Excel. We recorded the type of MBIs, type of intervention in the control group, authors, year of publication, number of participants, type of study, age and gender of participants, cortisol measurements and, finally, the main results of each study.

No statistical analysis or meta-analysis was performed due to the high heterogeneity among studies. Thus, the data were only descriptively analyzed.

## 3. Stress and the HPA Axis

Stress is a natural response to environmental disturbances; however, an adequate response to any acute stressor followed by the completion of that response is essential for maintaining survival [35,41].

Although stress is considered to be a health state that has multiple negative components (related to social life, work, relationships and family, among others), it must be considered that the (acute) activation of the metabolic processes involved in the response to stress allows an individual to adapt and respond to the different environmental perturbations that can alter homeostasis [39,41,42,43].

However, when disruptors have a chronic presentation pattern or are sustained over time, they have the opposite effect because they induce cellular damage and have been associated with outcomes such as depression, anxiety, post-traumatic stress, multiple metabolic and vascular diseases, chronic inflammation, autoimmunity and addictions [42,44,45,46].

### The HPA Axis and Stress Response

**Figure 2:**
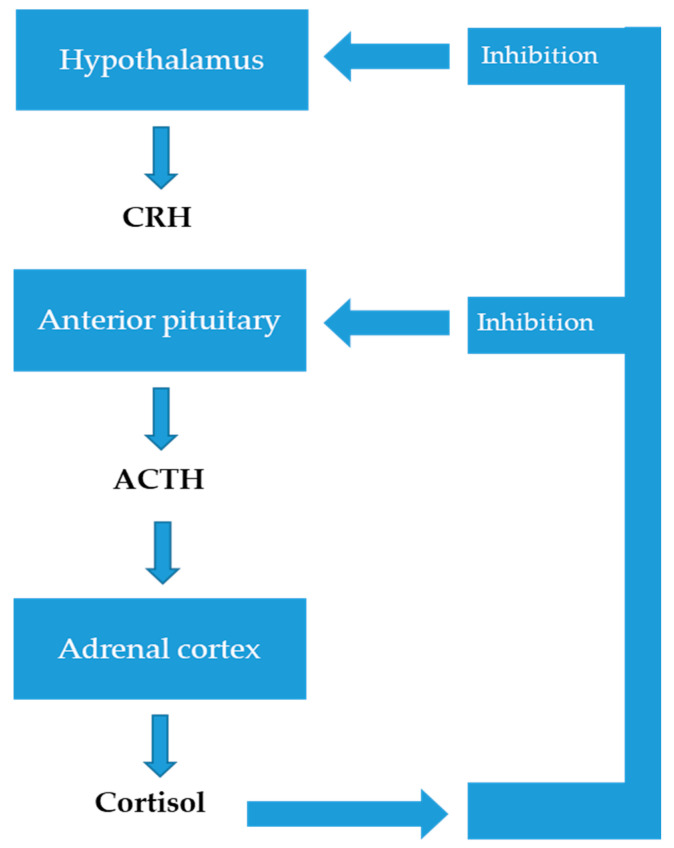
The hypothalamic–pituitary–adrenal axis, which describes the interaction between the hypothalamus, pituitary gland and adrenal glands. The main function generally attributed to the HPA axis involves the body’s reaction to stress (see text for more details).

The HPA axis is composed of three primary structures: the paraventricular nucleus of the hypothalamus (PVN), which integrates and regulates neuro-humoral stimuli to induce the activation of cells that synthesize and secrete the hormone-releasing, corticotropin-releasing hormone (CRH); this stimulates the anterior portion of the pituitary gland to synthesize and secrete the adrenocorticotropic hormone (ACTH). ACTH acts on the cells of the zona fasciculata in the adrenal cortex, which then synthesize and secrete glucocorticoids (especially cortisol) [47,48,49] (Figure 2).

Cortisol has a circadian rhythm that is regulated by a central pacemaker located in the suprachiasmatic nucleus of the hypothalamus. Physiologically, cortisol levels reach their lowest level around midnight, while the highest level is observed in the morning (8–9 a.m.) before decreasing throughout the day and increasing again in the afternoon [50,51].

The HPA axis is regulated by a feedback process that allows the basal homeostatic state to be restored; if we consider that the response of the HPA axis to environmental disturbances is adaptive, then dysregulation at any level of the three structures of the HPA axis can cause undesirable health outcomes [52].

If the HPA axis remains activated, the synthesis and secretion of cortisol increase, so the exposure of multiple tissues to the effects of this hormone also increases, (potentially) inducing allostatic overload and tissue damage [53].

The activation of the HPA axis has been shown to be related to an increase in cardiovascular reactivity to cortisol in individuals with stress and is associated with a higher risk of arterial hypertension, the calcification of coronary arteries, an increased heart rate, decreased heart rate variability, a high body mass index, adiposity, diabetes mellitus, a low-grade chronic inflammatory response, oxidative stress, endothelial dysfunction, atherogenic dyslipidemia and, ultimately, a high risk of cardiovascular and cerebrovascular outcomes [54,55,56,57,58,59].

## 4. Evaluation of Cortisol Levels, Principles and Utility

Clinically, in individuals with suspected endogenous hypercortisolism (hyperplasia or tumors producing CRH, ACTH or cortisol) or with adrenal insufficiency, the measurement of cortisol (in urine, saliva and blood) with suppression or stimulation tests, respectively, is part of the initial approach in diagnostic evaluation [60,61,62,63,64].

However, the HPA axis needs to be evaluated very differently in individuals with stress because such individuals have an undamaged HPA axis (in the absence of conditions that could alter its functioning, such as tumors, diseases, surgeries and the use of medications) [65,66,67].

Several ways to assess cortisol levels in individuals with stress have been proposed, which are summarized below.

### 4.1. Basal Cortisol

The circadian distribution of cortisol can be helpful in the study of individuals with endogenous hypercortisolism because, in this clinical scenario, the circadian pattern is largely lost (with persistently high values observed, especially around midnight) [68,69].

Similarly, measuring basal cortisol allows the identification of patients with adrenal insufficiency, and although there is controversy regarding the cutoff points for establishing the functioning of the HPA axis (based exclusively on the measurement of cortisol, between 8 and 9 a.m.), it is accepted that a value >18 µg/dL practically rules out the diagnosis, while a value <3.0 µg/dL confirms it. Additionally, the concomitant measurement of ACTH also allows us to identify whether the insufficiency originated in the adrenal gland or at the hypothalamus–pituitary level [70,71,72].

Although the measurement of basal cortisol allows for the functioning of the HPA axis to be identified in a global, simple and low-cost manner, this test has not been validated for the evaluation of patients with chronic stress.

### 4.2. Free Cortisol in 24 h Urine

Measuring the free cortisol in 24 h urine could be considered a strategy for the evaluation of the HPA axis, but it must be noted that the validity of this test depends on whether kidney function is normal because, when the filtration rate is <60 mL/min, cortisol excretion begins to decrease; additionally, excessive or insufficient urine collection can bias the results, so creatinine excretion must be measured at the same time (which increases costs) [73,74,75].

In individuals with endogenous hypercortisolism (with a high pretest probability), an elevated concentration of free cortisol in 24 h urine increases the positive predictive value of having the disease (and the test demonstrated its usefulness in this clinical scenario, although it is generally necessary to perform the test several times to demonstrate the consistency of the results) [76,77].

However, the test has not been validated for use in evaluating patients with suspected adrenal insufficiency or patients with chronic stress; therefore, its usefulness has not been demonstrated in these individuals.

### 4.3. Hair Cortisol

Cortisol is a lipophilic substance that can be obtained from the hair of the scalp because it moves from the circulation to the medullary region in the center of the hair. The sources of cortisol on the hair surface include both sweat and sebaceous glands, and it has been established that these sources probably reflect the state of free cortisol rather than protein-bound cortisol. For this test, it is necessary to take a hair sample between 1 and 3 cm in length. The importance of hair cortisol lies in the fact that it averages cortisol levels over the last 3 months (reflecting a more meaningful marker than basal cortisol or free cortisol in 24 h urine) [78,79,80,81].

Hair cortisol (in patients with chronic stress) has recently been described as a useful alternative biomarker for evaluating the HPA axis, with the advantage of it being free of certain factors that can alter serum, plasma or urine cortisol measurements; however, this test is affected by factors such as race, hair growth rate, and the use of dyes, shampoo and bleaching; additionally, there are no standardized measurement units available for its reporting, nor have normality values in different age groups been established [82,83,84,85].

### 4.4. Salivary Cortisol

Due to the circadian pattern of cortisol, an attempt has been made to identify alternative means of measuring cortisol; among them, measuring cortisol in the saliva stands out. This approach involves the collection of several saliva samples at various times of the day to identify the different points that make up the circadian pattern of the hormone [86,87].

The majority of the components of saliva are blood derivatives and cortisol diffuses to the salivary glands (independently of salivary flow), thus facilitating the evaluation of the circadian rhythm and, especially, the usual secretion peak in the morning hours. Because there is no bound cortisol in saliva, its levels are considered to be independent of blood cortisol levels [88,89].

Several advantages of performing this test include the fact that the sample collection is noninvasive, which reduces the risk of cortisol levels being overestimated. In addition, sample collection can compensate for sudden changes in the levels of free cortisol in 24 h urine or in blood cortisol. Furthermore, the measurement of salivary cortisol levels (at midnight) is highly sensitive and specific for the diagnosis of Cushing’s syndrome and is currently one of the first-line tests for the diagnostic evaluation of patients with this syndrome; however, its usefulness in other clinical settings has not been validated [90,91,92].

### 4.5. Cortisol Awakening Response (CAR)

Another way to assess HPA axis activity (via salivary cortisol) is by measuring cortisol within the first 60 min after waking up, i.e., the cortisol awakening response (CAR) [93].

This parameter combines the characteristics of a reactivity index (awakening response) with aspects related to circadian regulation (occurring at approximately the same time every 24 h); therefore, the CAR represents a strong increase in cortisol levels during the first 30 to 45 min after morning awakening [93,94].

The CAR is a normal expression of the circadian physiology of the HPA axis; therefore, deviations in its pattern may be a marker of a maladaptive neuroendocrine response; these characteristics of the CAR have led it to be considered one of the most common tools for evaluating the HPA axis in individuals with chronic stress [95,96].

## 5. MBIs and Stress

There are numerous strategies that have been shown to be effective for stress management; in general terms, almost all of them complement each other and involve changes toward “healthy” lifestyles such as increasing physical activity, improving nutrition and improving the quality of sleep; more recently, approaches that can improve cognitive and emotional appearance have been considered [97].

MBIs have aroused widespread clinical interest in recent decades. In this type of approach, structured interventions allow individuals to learn about meditation, body scanning techniques and physical exercises inspired by yoga, which means that these individuals can process their emotions, thoughts and sensations to the extent to which they emerge [98,99,100] (Table 1).

It has also been found that mind–body strategies, meditation-based interventions and MBIs can reduce cortisol levels; in fact, meta-analytic evidence suggests that stress management interventions are effective at changing cortisol levels in healthy adults and in other clinical settings [101,102,103,104].

**Table 1:** Summary of results regarding commonly used MBI programs in different studies that evaluate their effectiveness regarding different clinical and/or biochemical outcomes, as well as their definitions [105,106,107,108,109,110,111].

MBI Programs Most Frequently Used in the General Population
Mindfulness-based stress reduction (MBSR) The standard eight-week course of MBSR consists of an instructor delivering group instruction for 2.5 h/week (consisting of meditation practice, group discussions and mindfulness skill-building activities), a single half-day meditation retreat and daily practice of 30–45 min 6 days per week. The goal of this course is to integrate mindfulness in the participant’s daily life. Between weeks six and seven, an intensive mindfulness day is held, where participants practice about 7 h of different meditation practices.
Mindfulness-based cognitive therapy (MBCT) MBCT is an eight-week course program with weekly group meetings; participants meet together as a class (with a mindfulness teacher) for two hours a week for eight weeks, with an additional all-day session between weeks 5 and 7. The main ‘work’ is completed at home between classes.
Heart and mind—Mindful meditation program (HMMMP): healing the heart and mind This program is an eight-week course with weekly group sessions of 2 h and combines the main elements of MBSR as well as elements of cognitive therapy. During the duration of the course, participants are obligated to practice 30 min a day with audio recordings
Mindfulness-based relapse prevention (MBRP) MBRP is an intervention that integrates mindfulness meditation with traditional relapse prevention techniques. It has three main components: formal mindfulness practice, informal practice and coping strategies. The MBRP program has a total of 16 h divided into eight weekly sessions (2 h each) but can be adapted according to the target population and the researchers’ goals, during which participants with a history of substance use disorders learn formal mindfulness practices as well as how to integrate them into their daily lives.
Mindfulness-based stress reduction for teenagers (MBSRT) MBSRT is based on secular adaptations of mindfulness practices with roots in Eastern meditation traditions and is also strongly influenced by MBCT; it is a four-week program with eight semiweekly group sessions, especially adapted for adolescents (13–18 years). The goal of the program is to direct attention to what nourishes and fills us (rather than what drains and depletes us).
Low-dose mindfulness-based stress reduction (LDMBSR) This program consists of providing group instruction for 60 min, once a week (for 6 weeks), incorporating aspects such as breathing, relaxation, body scans and gentle yoga movements as a means of facilitation towards a meditative state. The main objective of the technique is to improve the participants’ ability to pay attention to their internal and external experiences moment by moment. This program is oriented toward managing local stresses commonly experienced by healthy working adults.
Mindfulness-based stretching and deep breathing exercise (MBX) MBX consists of 16 semiweekly group trainings of 1 h. The intervention focuses on balancing and stretching movements combined with deep breathing and mindfulness. During the sessions, participants are instructed to pay attention to the flow of each movement in the present moment, focusing on the conscious regulation of breathing. Over the course of 8 weeks, the intensity of the exercise increases, but the sequence of movements remains the same.
Mindfulness-based mind fitness training (MMFT) MMFT is designed to help individuals widen their window of tolerance to stress. This program is an eight-week course with weekly group sessions of 2 h and an additional intensive practice workshop of 4 h. Alongside the class sessions, participants are obligated to practice a minimum of 30 min of mindfulness exercises and self-regulation practices.
Primary care brief mindfulness practice (PCBMP) The PCBMP was designed to enhance emotional regulation and the cognitive reappraisal of stressors. It is a four-week program (90 min mindfulness-based meditation group sessions for four consecutive weeks led by an experienced trained MBSR facilitator). To support home practice, participants receive a copy of the book Full Catastrophe Living: Using the Wisdom of Your Body and Mind to Face Stress, Pain, and Illness and audio CDs containing prerecorded guided practice (5, 10, 30 and 45 min body scans and guided meditation, and 30 min standing and lying yoga) made by the facilitator to support home practice.
Mindful awareness practice for daily living (MAP) MAP is a six-week program with weekly group sessions of 2 h. During the duration of the course, participants are asked to complete daily meditation homework; the program starts with 5 min per day and the practice time gradually increases, with a reasonable goal of 20 to 45 min per day.
MBI—Workplace (MW) This program combines elements of mindfulness meditation, yoga and relaxation through music and involves three steps: an obligatory 2 h introductory session concerning mental health and mindfulness; participation in a 10-week workplace-adapted live online MBSR program; and a workshop on the further implementation of mindfulness in the companies of the selected employee representatives and managers.
MBI—Audio (MBIA) MBIA uses a 20 min audio guide version of the MBSR body scan. Participants are asked to practice daily for a duration of eight weeks.
Monitoring and accepting (MA) MA describes the mechanisms of mindfulness for cognition, affect, stress and health. This program is an eight-week-long course with weekly group sessions of 2 h and follows the standard MBSR protocol with a specific focus on monitoring and acceptance. Alongside the group sessions, participants are asked to complete daily 45 min home practices.
Mindfulness-based intervention apps (MBI-App) The smartphone-based MBI is a two-week long program with daily 20 min audio-guided practices and an additional 3 to 10 min of non-guided practice. It focuses on monitoring and accepting the body and emotions and is based on elements of MBSR.
MBI—Training (MBIT) This MBI is an eight-week course with weekly group workshop sessions of 1 h and contains elements of MBSR and MBCT. These brief and highly controlled interventions typically involve 20 to 30 min group sessions conducted by a trained meditation instructor. In addition to the weekly group sessions, it includes daily 30 min home practice sessions and mindful Wushu training sessions. The aim is to allow for the adaption of mindfulness into the participants’ competitive environment and sport practices.
Mindfulness-based art therapy (MBAT) MBAT is based on Kabat-Zinn’s mindfulness meditation and reflects on MBSR and the self-regulation theory. MBAT integrates mindfulness meditation skills and aspects of art therapy into an eight-week, gender-segregated, supportive group therapy format. The multi-modal design is intended to provide opportunities for both verbal and non-verbal expression, enhanced support and expanded coping strategies.
Mindfulness-based resilience training (MBRT) MBRT is an eight-week program (2 h sessions, with an extended 6 h class in weeks 1 and 7) integrating training in standardized mindfulness practices targeting factors that facilitate resilience with cognitive behavioral therapy (CBT) and psychoeducation. The general curriculum structure is modeled after the MBRP clinical protocol. The content and language have been altered to be more relevant, and there is emphasis on working with reactivity to stressors inherent to police work, including critical incidents; job dissatisfaction; public scrutiny; and interpersonal, affective and behavioral challenges.
Mindful parenting (MP) Mindful parenting is a six-week course with weekly group sessions of 1.5 h that includes typical mindfulness practices of the MBSR program, emphasizing the central role of intention (on purpose), attention (paying attention) and attitude (with openness and nonjudgment) in mindfulness and how these then facilitate “reperceiving” via four key mechanisms: self-regulation, value clarification, cognitive–behavioral flexibility and exposure.
Mindfulness matters (MM) MM is an eight-week course with weekly sessions of 1 h, especially designed for children between 5 and 8 years old. The program includes aspects such as awareness, acceptance and gratitude through storytelling and nuggets of age-appropriate theory. The exercises (mindful breathing, awareness of the senses and body scans) and mindful content (mindful movement, guided meditation, relaxation and visualization) can be completed at home and school. The course includes short mindful meditation practices as well as methods like storytelling.
Acceptance and commitment therapy (ACT) The ACT interventions focus on two main processes: developing acceptance of unwanted private experiences that are out of one’s personal control, and commitment and action toward living a valued life. The ACT is an eight-week program with a total of six group sessions of 1.5 h. The sessions include mindfulness-based elements and focus on one’s values, committing to those values, and general acceptance and flexibility.
Brief mindfulness meditation (BMM) BMM is a three-day mindfulness course, during which participants practice 30 min of sitting meditation with an audio guide (audio-guided meditation focused on the body and breathing sensations) based on the standard sitting meditation of MBSR. Patients perform mindfulness exercises, during which they are instructed to observe each aspect of the current experience, such as thoughts and feelings, with acceptance and curiosity.
Characteristics of the MBI programs most frequently used for certain conditions and specific populations
MBI—Physicians (MBIP) MBIP consists of an intensive phase of eight weekly sessions of 2.5 h, plus an all-day session (7 h) between the sixth and seventh weekly sessions and a maintenance phase (10 monthly sessions of 2.5 h after the eighth weekly session). The all-day session is structured as a silent retreat in which participants participating in guided mindfulness practices are asked to remain silent for an entire day in a retreat center.
MBI—Smokers (MBISM) MBISM is mindfulness training for smokers, basically following the MBSR program. Participants are encouraged to apply nonjudgmental (moment-to-moment) awareness to strong emotions or thoughts, which often involve cravings, negative affects or withdrawal symptoms. Subjects are instructed to practice mindfulness throughout the day, including during meals, social interactions and times associated with situational threats to return to smoking.
MBI—Dementia caregivers (MBIDC) MBIDC is a six-week program with weekly group sessions of 1.5 h and combines elements of MBSR and MBCT. The MBIDC has shortened sessions (1.5 h each) compared to the traditional MBSR 2.5 h sessions, to better accommodate caregivers’ needs. The program is adapted to fit the specific needs and difficulties of dementia caregivers and focuses on different aspects of stress and promoting self-care as well as feelings of competence and mastery.
MBI—University (MBIU) This MBIU lasts for 3 months, is embedded in a standard curriculum course for medical students and includes a one-day introduction, four 3 h group sessions and a final 2 h closing session. Students learn about typical elements of the MBSR program and are asked to practice them daily.
MBCUL—Mindfulness-based coping with university life MBCUL is an eight-week meditation-based program that aims to introduce students to the concept and practice of mindfulness meditation (eight consecutive weeks in the campus dance studio, one evening per week, for 1.5 h starting at 18:00 h).
MBI—Students (MBIST) MBIST consists of six meetings; the first and last meetings last for 1 h 15 min, while the remaining ones last for 35 min. It is a seven-week program. In each meeting, the participants fill out a self-report form on meditative practices, where they record the number of practices they have executed since the last meeting they attended. This program is adapted for students and focuses on flexibility, self-compassion and empowerment to optimize well-being and resilience.
MBI—Teachers This MBI is a 16-week program with one monthly 1.5 h group meeting, especially designed to reduce stress and burnout among teachers. The teachers develop personal mindfulness practices as well as mindfulness practices to be applied within their teaching role (e.g., mindfulness of breathing, mindfulness of body sensations and mindful listening). Alongside the group sessions, participants fill out workbooks for their home practice.

## 6. Studies Evaluating Possible Associations Between MBIs and Cortisol Levels

Several studies have shown that MBIs can significantly modify cortisol levels (in different populations with various underlying pathologies), especially MBSR and MBCT. However, other studies have shown an increase in cortisol levels in participants with low basal levels (prior to MBIs), suggesting that, in this subgroup of individuals, MBIs tend to normalize cortisol levels over time.

There are also other studies that have not demonstrated significant changes in cortisol levels after MBIs; therefore, the scientific literature evaluating the effects of mindfulness on cortisol levels has been inconsistent and not always reproducible [112].

Although each of the studies selected for this systematic review was independently examined (according to the JBI Critical Appraisal Checklist), we still found limitations in several of the studies analyzed, such as a lack of information on how the randomization process was carried out, in addition to the fact that there were incomplete data on the procedures for allocation concealment and the blinding of outcome assessments.

In addition, most of the studies had small sample sizes and also did not report data on the characteristics of participants who were lost to follow-up.

A common finding in several of these studies is that no description was made of the “control” group (and when they were, the interventions that were carried out were not always well defined or specified).

These characteristics prevented a quantitative synthesis and meta-analysis from being carried out with the studies chosen for the analysis.

### 6.1. Mindfulness and Cortisol Levels: Studies with Significant Changes

Several studies have shown that significant changes in cortisol levels occur after MBIs. These changes have been found in individuals with multiple health conditions and in different settings.

In summary, the participants in these studies included the following categories: substance abuse, smoking, college or school professors, college students, college workers (not professors), people with PTSD (nurses and veterans), breast cancer patients and survivors, law enforcement officers, basketball players, disadvantaged families, novice meditators, health workers (nondoctors), people with overweight–obesity, diabetes mellitus, ulcerative colitis and, finally, healthy individuals.

Of the 25 studies identified, only 3 studied populations that were predominantly male; 3 of the studies were conducted exclusively in women, 1 study did not report the sex of the participants and only 1 of the studies had the same proportion of subjects with respect to sex.

The MBIs consisted of MBSR (or modified MBSR), MBCT or a combination of the two, while the comparison groups (controls) were as follows: wait-list, neutral clay task, supportive expressive therapy, stress management course, stress management seminar, time/attention control, treatment as usual, usual care, heart rate variability–biofeedback, vacation retreat, individuals with the freedom to quit smoking, walking exercise, patient education, active control, nonmanipulated inactive group, web-based student compass program, acceptance and commitment therapy, and health enhancement programs. Some studies did not have or report a control group.

Cortisol levels were evaluated in hair (3 studies); in 24 h urine, along with ACTH levels in blood (1 study); in plasma (2 studies); in serum and saliva (1 study); and only in saliva (18 studies).

Hair cortisol levels were evaluated before the start of the MBI, at baseline and post-intervention and after the four-month follow-up. The serum ACTH (blood) and free cortisol levels in 24 h urine were measured at baseline (visit 1, pretreatment visit), after 8 weeks (visit 2, weeks 9–12) and at 6- and 12-month follow-ups (visits 3 and 4).

The plasma cortisol levels were assessed on the first and third days of training between 8 and 9 a.m. after 45 min of rest or before and after the intervention. The serum cortisol concentrations were evaluated in the morning.

The salivary cortisol measurements included parameters such as the CAR, the baseline (and post-intervention) cortisol concentration, a single sample after waking up, the daily cortisol concentration, several measurements on the same day for several days, the cortisol slope, the cortisol levels between 7 and 10 a.m. and the AUC of cortisol.

The results of the salivary cortisol measurements were discordant; for instance, awakening (post-intervention) levels were significantly lower in some studies but greater in others. The baseline salivary cortisol concentration was also greater in the control groups than in the intervention groups, and there were greater reductions in CAR over time in the intervention groups.

An increase in CAR after awakening was also demonstrated in the intervention groups, and some studies showed a greater increase in basal cortisol levels in the intervention groups post-therapy.

The diurnal cortisol slopes were significantly more negative following interventions such as SET and MBCR (compared to SMS), and a significant increase in the cortisol slope was observed from baseline to post-intervention in participants assigned to SMS.

Other studies showed a significant decrease in morning cortisol in the control group. Some studies found no differences in the AUC of cortisol in the groups evaluated, while others did.

On the other hand, there was a significant difference in ACTH levels (increase) over time between participants in the intervention groups, with no significant changes in free cortisol in 24 h urine.

Similarly, the plasma cortisol levels were significantly lower in the intervention and control groups (after the intervention). Finally, the average hair cortisol concentration decreased significantly in all participants; for example, some studies showed a significant reduction in the intervention groups, while others found no differences between the groups evaluated (Table 2).

### 6.2. Mindfulness and Cortisol Levels: Studies Without Significant Changes

Contrary to expectations, some studies did not find significant changes in cortisol levels among individuals who had undergone MBIs. These studies evaluated subjects with the following conditions or inclusion criteria: university students, healthy participants, subjects with recurrent depression, university faculty and staff, resident physicians, nurses, healthcare professionals, community-dwelling caregivers, experienced meditators and students exposed to exam stress.

Among the 10 studies evaluated, all involved a predominance of females. The MBIs consisted of MBSR (or modified and adapted MBSR), MBCT and combinations of MBSR and MBCT, while the comparison groups (controls) were as follows: wait-list, yoga, BMV, TAU, education control, mindfulness, relaxing music, nonintervention, education class, pragmatic control, meditation naïve subjects and support as usual.

Cortisol levels were evaluated in saliva (eight studies); hair (one study); and blood (one study). In the study evaluating hair cortisol, hair samples 1 cm in length and 3 mm in diameter were taken at four on-site appointments.

In the only study that assessed blood cortisol levels, only one sample was taken, but the time at which it was taken was not reported.

Salivary cortisol measurements included parameters such as the CAR, baseline (and post-intervention), the cortisol slope, a single sample after waking up, noon and bedtime, daily cortisol, several measurements on the same day for several days, and the AUC of cortisol.

In these studies, the MBIs did not lead to significant differences in cortisol levels (Table 3).

## 7. Discussion

Several hypotheses can explain the associations between MBIs and cortisol levels, and the evidence from the studies listed in Table 2 and Table 3 indicates discordance in the results. Thus, two main hypotheses can be proposed:

### 7.1. Hypothesis 1. MBIs Impact Cortisol Levels

Neuroplasticity is a phenomenon that underlies the different changes in neuronal pathways and neuronal synapses. In addition, neuroplasticity plays a predominant role in neuronal communication with the endocrine system, thus allowing for multiple adaptive responses to different stimuli. Cortisol intervenes in the process of neuroplasticity and induces neuroplastic changes at the hippocampal level [44,148].

Three major large-scale brain networks have been described: the salience network (SN), central executive network (CEN) and default model network (DMN). Under conditions of stress, a sustained increase in cortisol levels can affect the function of neuronal structures of the DMN, including the hypothalamus [149,150].

In this order of ideas, a significant change in cortisol levels has been demonstrated to be a consequence of greater executive control promoted by the nonjudgmental awareness of present-moment experiences cultivated during the practice of MBIs. This approach is possible because refined openness and awareness allow for subtle changes in affective states to be detected with greater flexibility, which in turn reduces habitual rumination tendencies, thereby improving the adaptive regulation of emotions [150,151].

It has also been considered that MBIs mainly affect three aspects: the control of attention, the regulation of emotions and self-awareness. Each of these aspects is susceptible to modification after repeated and continuous mindfulness practice, inducing changes in the functional and structural aspects of various brain structures, such as the anterior cingulate cortex (ACC), the prefrontal cortex (PFC), the posterior cingulate cortex (PCC), or areas such as the insula, amygdala and the striatum [151,152].

The neuroplastic effects of meditation on the DMN suggest that functional connectivity changes and differs between people who practice MBIs or meditation-based interventions (compared to nonpracticing subjects); these connections are involved in the phenomena of emotional processing and evaluation in areas that involve the PFC, PCC and the dorsolateral prefrontal cortex (DPC) [152,153].

These structures are part of the limbic system (or multiple circuits of the limbic network) and the hypothalamus is considered a group of anterior diencephalic nuclei (that are anatomically located in the center of the limbic system) [154].

The limbic system is classically considered to be activated in the presence of stress (as an adaptive response to stress); thus, in situations involving stress, the activation of structures such as the amygdala has been associated with elevated levels of cortisol. Therefore, the amygdala plays a facilitating role in the responses of the HPA axis to different stressful stimuli [154,155].

This effect is mediated by neurotransmitters such as norepinephrine (NE), its α-1 receptor, serotonin (5-HT) and its 5-HT2 receptor. The neuroendocrine effects of NE in the hypothalamus are mediated by intrahypothalamic glutamatergic neurons. Furthermore, the amygdala facilitates the release of 5-HT from the paraventricular nucleus of the hypothalamus in response to stress and is capable of attenuating the negative feedback exerted by cortisol (probably reducing glucocorticoid receptors in the hippocampus, thus facilitating the activation of the HPA axis), with a greater release of CRH–ACTH [156,157,158].

**Figure 3:**
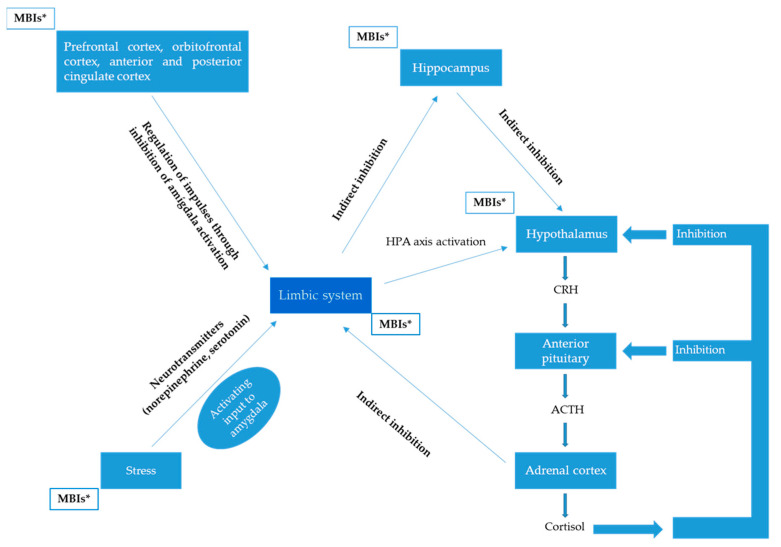
Under stress conditions, a sustained elevation of cortisol levels can affect several brain structures, including the hypothalamus. MBIs can induce changes in the functioning of brain areas such as the anterior cingulate cortex, the prefrontal cortex, the posterior cingulate cortex and the limbic system. Furthermore, MBIs can modify cortisol levels by inducing functional and structural changes in brain areas directly related to CRH-ACTH secretion (see text for more details). * Mindfulness-Based Interventions.

In this way, MBIs could modify cortisol levels by inducing functional and structural changes in brain areas that are directly related to CRH–ACTH secretion (Figure 3).

From another point of view, we cannot ignore the fact that most of the studies had small sample sizes, there was no “masking” of the groups and confounding or effect-modifying variables (interaction) were not taken into account.

These aspects may influence the results described, and the findings actually suggest a type I error (indicating a spurious relationship between MBIs and cortisol levels). Furthermore, there was a high risk of “interviewer bias”, as there was no masking of the people who trained the subjects in the MBI groups [159,160].

Similarly, “differential classification error” cannot be ruled out because there could (potentially) have been greater sensitivity in the definition of the subjects in the intervention groups (compared to the subjects in the control groups), which could have biased the association away from the null hypothesis [161].

Additionally, a possible “medical surveillance bias” can be suggested because the identification of the outcome, in this case, cortisol, was not independent of knowledge of the exposure (inclusion criteria), which could have led to greater surveillance and follow-up in the intervention group (in fact, several studies had the so-called wait-list as a control group), which could have led to a finding of a positive effect of MBIs on cortisol levels [162].

### 7.2. Hypothesis 2. MBIs Do Not Impact Cortisol Levels

There are several explanations of this hypothesis. For example, some of the studies were carried out on healthy people, which may indicate that the interventions have no effect on cortisol in people with no diagnosis of depression, anxiety or stress; in fact, some studies found a beneficial effect of MBIs only in individuals with underlying conditions (depression, anxiety, stress, etc.) [163].

The lack of effects of MBIs on cortisol levels could also be explained by the time that the study participants spent on the MBIs because, in most of the studies, although MBI strategies were used, they did not all involve a unified protocol or program (in several studies, modified MBSR or MBCT protocols or programs were implemented), which could explain the differences between studies that show changes in the level of cortisol and other studies that do not.

This finding contrasts with the findings of studies that showed a change in cortisol levels after MBIs, which generally had a basic design in which the original MBSR or MBCT protocol was used, with some studies involving slight modifications to the original protocol but with very similar durations. In the studies that did not show modification of cortisol levels (after the MBIs), this aspect may be secondary to a “temporal” bias given the time spent on the interventions in those studies [164].

Additionally, the studies used tissues such as saliva, hair, blood (or its derivatives, plasma or serum) and urine as methods for measuring cortisol; based on this, it should be considered that cortisol shows moderate to high intra- and interindividual variability, which can be explained by environmental factors; for example, the intraindividual variability is higher among patients with depressive disorders than among healthy controls [165].

Additionally, some “erratic” patterns have been described in subjects with depressive episodes of greater severity or with recurring episodes, which could reflect greater deregulation of the HPA axis in these individuals [166].

Likewise, it has been noted that the HPA axis response decreases or habituates when there is repeated or continuous exposure to a stressor (homotypic stressor); however, if the HPA axis faces a new stressor (heterotypic stressor), the response tends to be normal or “facilitated”, which may explain why some individuals with a homotypic stressor may have an attenuated HPA axis response (with reduced absolute levels of cortisol), such as individuals with fibromyalgia or chronic fatigue [167].

Other factors that modify the HPA axis response include the sleep–wake cycle (quality of sleep and insomnia, among other factors), along with the states of rumination and worry in the days prior to taking the cortisol sample, including waking up in the dark or daylight and measuring cortisol on weekdays or weekends [168,169,170,171,172].

Furthermore, a possible seasonal effect on the activity of the HPA axis was found in some studies, such that “month by month” changes in cortisol levels were identified at the population level; for example, in some studies, higher concentrations of cortisol were described in the spring and lower levels were observed in the summer. One study even showed that the highest levels of cortisol (morning and evening) occurred during the months of the year with less sunlight [173,174].

Each of the factors that modify cortisol levels can act as a confounding variable, and none of the studies adjusted for these factors or performed stratified analyses based on these potentially confounding variables. Furthermore, potential interactions between variables were not considered (a fundamental aspect when analyzing studies that carry out some type of intervention) [175].

Moreover, the majority of the studies used an uncontrolled design and had small sample sizes (which increases the risk of a type II error, especially if the MBIs are expected to have a subtle effect on the level of cortisol) [176].

Additionally, “nondifferential classification errors” cannot be ruled out, especially in studies that used two categories (an intervention group and control group); in this sense, this error may appear when the exposure (inclusion criteria) is the same for the subjects in both study groups, which tends to bias the association toward the null hypothesis [177].

It should also be considered that when differences are detected between different groups with different interventions, these differences may be affected by the presence of a low “signal-to-noise ratio”; that is, in the group classified as a control (or nonintervention), the outcome showed a change similar to or greater than that in the intervention group, potentially affecting the results and the expected association [178].

Consequently, it is likely that the forms of cortisol measured in the different studies do not reliably determine the real state of the HPA axis; in fact, there is no standardization of these tests or reference ranges to identify alterations in the HPA axis beyond the diagnosis of endogenous hypercortisolism or adrenal insufficiency; therefore, it is likely that the studies that did not show an effect of MBIs on cortisol levels were a reflection of laboratory tests that did not reliably evaluate the HPA axis under the inclusion criteria established in the studies described [179,180].

Our systematic review is not without limitations; first, the studies were very heterogeneous with respect to the inclusion criteria, the type of MBIs used and the definition of the outcome to be evaluated.

Second, it should also be noted that more than half of the studies included in this review had fewer than 20 participants in the intervention group (and in some of them the number of subjects evaluated was not even described). The results of a study with a small sample size may not be applicable to all interested people (loss of external validity) and it is also less likely that the real effects of an intervention will be detected, especially the rarer and less striking effects (of smaller magnitude), making it difficult to find significant differences between participants and increasing the probability that the results of the study are due to chance.

Third, several of the studies were non-randomized; randomization is the ideal elective method to achieve comparability between groups, as it strengthens the statistical tests used, reduces selection bias and attempts to protect against other types of bias, fundamentally that of confusion (its main purpose).

Fourth, the types of intervention used in the control group were very diverse, and the different studies established multiple ways of measuring cortisol, which made it difficult to carry out a more robust and specific analysis of the results presented.

Finally, we suggest that future studies consider assessing the HPA axis using expanded and complementary laboratory strategies, such as insulin-mediated hypoglycemia stimulation tests; CRH stimulation tests; or a combination of hair, saliva and/or blood measurements. Additionally, MBI strategies should be standardized, with specific definitions for different populations (with larger sample sizes and well-defined randomization tools), ensuring a lower risk of bias and confounding factors. If present, the identification and intervention strategies used should be presented and described in order to determine whether MBIs really significantly modify the results associated with different health outcomes.

## 8. Conclusions

There is a widespread belief that MBIs positively modify various outcomes associated with stress; however, some of the available evidence is circumstantial and based on studies with small sample sizes and different inclusion criteria, intervention strategies and follow-ups. Likewise, the evaluation of cortisol as a stress marker has also been based on the diagnostic concepts of individuals with endogenous hypercortisolism or adrenal insufficiency; additionally, the reference values of cortisol measurements in individuals who undergo interventions with MBIs have not been standardized, which may partly explain the discrepancies in the results of the studies carried out.

We can biologically explain the association between stress and cortisol levels in addition to the ways in which MBIs can modify neurometabolic responses to stress and cortisol levels. However, the included studies were not robust enough in their designs to draw definitive conclusions.

Standardized cortisol collection methods (such as hair cortisol) seem to be a tool that, in the future, could allow for a better assessment of cortisol status in individuals receiving MBIs. However, this must be demonstrated in more longitudinal studies involving multiple measurements of cortisol at various times of the day (and night), including various measurement methods (in various tissues).

We believe that, based on the current evidence, MBIs could have a significant effect on cortisol levels, although the magnitude of these effects is likely to be small.

## Figures and Tables

**Table 2:** Summary of the results of studies that demonstrated significant changes in cortisol levels, with MBIs (n = 25).

Author, Year [Ref.]	Subjects: Age in Years (y) and Gender; M = Male; F = Female; Population (P)	Intervention Group (n)	Intervention in Control Group (n)	Type of Intervention	Cortisol Measurements	Results
Marcus, 2003 [113]	Mean age: 33.4 y. Gender, F: 14.3. P: therapeutic community for substance abuse	MBSR (21)	Not reported	Original MBSR program	Salivary cortisol: 0, 30, 45 and 60 min after awakening (CAR) pre- and post-intervention	The increase in cortisol upon awakening after the intervention was significantly less than that before the intervention
Klatt, 2009 [114]	Mean age: 44.95 y. Gender, F: 75%. P: full-time university professors and staff	LDMBSR (22)	Wait-list (23)	Similar to the MBSR original program. The duration of the weekly meetings was reduced from 2.5 to 3 h to 1 h, the daily 45 to 60 min sessions of meditation were reduced to 20 min of meditation/yoga, and the full-day retreat within traditional MBSR was omitted. The wait-list control group was offered LDMBSR	Salivary cortisol: Baseline, established from 2 consecutive days of sampling conducted 20 min after waking, at 1:00 p.m. and 10:00 p.m.; once per week on the same day of the week during each week of the intervention; and again for 2 days at 1 week after the completion of the intervention	The baseline salivary cortisol was higher in the control group compared to the LDMBSR group
Daubenmier, 2011 [115]	Mean age: 40.9 y. Gender: only women. P: overweight and obese	In the intervention groups, a preliminary, novel intervention was developed, drawing on the components MBSR, MBCT and MB-EAT (24)	Wait-list (23)	The intervention program consisted of nine 2.5 h classes and one 7 h silent day of guided meditation practice after class 6. Those in the wait-list group were offered the mindfulness program after the completion of all the post-treatment assessments	Salivary cortisol and serum cortisol: For salivary cortisol, the CAR and cortisol slope were measured; the participants collected saliva samples at home on 4 days pre- and post-treatment. Serum cortisol was evaluated in the morning	The treated participants showed a significant trend of greater reductions in CAR over time compared to the control group
Beerse, 2020 [116]	Age1; 9.1 y (in M). Gender, F: 85.7%. P: full-time students at a public university	MBAT (41)	Neutral clay task (36)	2.5 h (5-week intervention covering 10 modules, each lasting 15 min, the first and last being in person and facilitated)	Salivary cortisol (samples taken pre- and post-intervention at a time slot between 7 and 10 a.m.)	Significant reductions in salivary cortisol were observed in the MBAT group
Matousek, 2011 [117]	Mean age: 55.9 y (range: 28–72 y). Gender: only women. P: breast cancer Patients	MBSR (33)	There was no control group	Original MBSR program	Salivary cortisol; the CAR was assessed on 3 days pre- and 3 days post-MBSR	A significant effect on the CAR was found, with cortisol levels showing a prolonged increase after awakening in the post-MBSR assessment period
Kim, 2013 [118]	Mean age: 45.73 y. Gender, F: 96.55. P: nurses with PTSD	MBX	Control group or participants without PTSD (BASE group)	MBX	Salivary cortisol was measured at baseline and weeks 4 and 8 in three groups. For the MBX group, serum cortisol was also measured at week 16	Cortisol levels showed a significant difference over time between groups. At post-intervention week 8, the basal serum cortisol concentrations were significantly higher in the MBX group than the control group. A significant increase in basal serum cortisol in the MBX group post-intervention was shown
Bergen-Cico, 2014 [119]	Mean age: 48 y. Gender, F: 10% P: veterans with post-traumatic stress disorder (PTSD)	PCBMP	TAU (21)	PCBMP Participants in the TAU group continued to receive the typical primary care treatment for veterans presenting with similar PTSD and subthreshold PTSD	Salivary cortisol: 5 saliva samples each day at specified times for 2 consecutive days for a total of 10 samples	Veterans completing 4 mindfulness-based meditation sessions significantly reduced their CAR, and showed a significant cortisol AUC increase compared with TAU participants
Carlson, 2013 [120]	Mean age: 54.85 y. Gender: only women. P: distressed survivors of breast cancer	MBSR (113)	Two groups: SET (104) and SMS (54)	Original MBSR program. In the SET group, the program consisted of 12-weekly group sessions of 90 min each. The minimal-treatment control condition group was a 1-day (6 h) didactic SMS	Salivary cortisol: participants collected saliva samples four times a day for 3 days before random assignment (30 min after waking and at 12:00 P.M., 5:00 P.M. and bedtime)	The diurnal cortisol slopes were significantly more negative after SET and MBCR (compared with SMS). A significant increase in the cortisol slope from baseline to post-intervention in SMS was observed
Brinkmann, 2020 [121]	Age: 43.27 y (in M). Gender, F: 71.2%. P: healthy participants	MBIs (19)	HRVB (23) or wait-list (27)	In all 3 groups: 36.6 h (6-week intervention consisting of 4 consecutive half days and 30 min daily self-guided training for 6 weeks). Two booster sessions in weeks 1 and 3	Salivary cortisol (one sample after waking; AM)	Cortisol changed significantly over time, with post hoc tests confirming a decrease in cortisol for all three groups between pre- and post-intervention
Christopher, 2018 [122]	Age, 44 y (in M) Gender, F: 10%. P: law enforcement officers	MBRT (31)	No intervention control (30)	MBRT	Three saliva samples taken at awakening and +30 and +45 min after awakening on three consecutive days pre- and post-intervention	MBRT participants experienced greater reductions in salivary cortisol
Lengacher, 2019 [123]	Mean age: 57.05 y. Gender: only women P: breast cancer survivors.	MBSRbc (167)	Usual-care group (155)	MBSRbc consists of educational material related to relaxation, meditation, the mind–body connection and a healthy lifestyle for BCS. Participants in the usual-care group were asked to refrain from practicing meditation, using yoga techniques or participating in MBSRbc for the duration of the 12-week study	Salivary cortisol: At baseline and 6 weeks, saliva specimens were collected for both groups. All samples were collected in the morning at the same time of day. For the MBSRbc group, saliva was also collected 20 min prior to and 20 min after the MBSRbc intervention session during weeks 1 and 6	Significant reductions in cortisol levels were observed with medium effect sizes pre- to post-MBSRbc session at weeks 1 and 6
Errazuriz, 2022 [124]	Age: 40.2 y (in M). Gender, F: 98.1%. P: non-physician health workers	MBSR (35)	SMC (34) or wait-list (36)	Original MBSR program. In addition, a day-long mindfulness retreat was offered between the sixth and seventh weeks	Salivary cortisol samples were collected three times in a day. The CAR and AUC of the cortisol were also evaluated during the day	MBSR decreased the CAR by 23%
Flook, 2013 [125]	Age: 43.06 y (in M). Gender, F: 88.89%. P: public elementary school teachers	Modified MBSR (adapted for teachers)	Wait-list	26 h (8 sessions over 8 weeks and a day-long immersion for 6 h)	Salivary cortisol: Three time points across the day were sampled to allow the analysis of both the diurnal cortisol slope and morning post-waking cortisol levels. Thirty minutes post-waking was chosen as the time point for the morning sample in order to assess the peak in the CAR	Both groups showed a marginally significant flattening of diurnal cortisol profiles over time. The control group showed a significant decrease in morning cortisol
Gardi, 2022 [126]	Age range: 18–67 y. Gender, F: 55.56%. P: healthy participants	Mindfulness meditation retreat (48)	Vacation retreat (47)	30 h (3-day retreat with 10 h of meditation per day)	Before (t0) and after (t1) the intervention, all the participants were assessed for salivary cortisol levels	At t1, a highly significant correlation between salivary cortisol levels and anxiety and stress perception scores was found
Goldberg, 2014 [127]	Age: 42.2 y (in M). Gender, F: 55.6%. P: smokers	MTS (10)	FFSE (8)	The MTS group received mindfulness instruction and the FFSE group received relaxation and CBT targeted to smoking cessation	Hair cortisol: A single 3 cm hair sample was obtained at the 1-month post-quit study visit. The 1 cm segment most proximal to the scalp was assayed to indicate the post-quit cortisol output. The second most proximal 1 cm segment, representing the month before the quit day, was not analyzed. The third most proximal 1 cm segment was assayed to indicate cortisol output the month before the intervention	Hair cortisol significantly decreased from baseline to 1 month after the quit attempt in the entire sample
Jedel, 2014 [128]	Mean age: 42.86 y. Gender, F: 56.36. Patients with ulcerative colitis in remission	MBSR (27)	TAC (28)	Original MBSR program. In the TAC group, the intervention was used to control for the time, support and attention that the subjects received in the MBSR group	The serum ACTH (blood) and cortisol in 24 h urine were measured at baseline (visit 1, pretreatment visit), post the 8-week course (visit 2, weeks 9–12) and at 6- and 12-month follow-ups (visits 3 and 4)	There was a significant difference in ACTH levels (increase) over time among participants in the MBSR group who had disease exacerbation (relative to those with exacerbation in the control group). There were no significant changes in urinary cortisol levels in the participants
Jung, 2015 [129]	Age < 70 y: 62.5%. Gender, F: 51.78%. Patients with diabetes mellitus	KMBSR (21)	Two groups: walking exercise group (18) and patient education group (17)	The KMBSR intervention lasted 8 weeks and was based on an audio CD, which facilitated mindful walking, eating, breathing and silent sitting meditation. In the walking exercise group, participants were encouraged to walk briskly for 30–60 min, 3–4 times per week. In the education group, patients were educated regarding improving knowledge about self-management behavior	Plasma cortisol, pre- and post-intervention	Plasma cortisol levels were significantly lower in the K-MBSR and walking groups subsequent to the intervention
Ho, 2020 [130]	Age: 38.75 y (in M). Gender, F: 96.08%. P: disadvantaged families	Family-based mindfulness intervention—parents and their children from 51 disadvantaged families (26)	Wait-list (25)	In the intervention group, the program lasted for 6 weeks and each session lasted 1.5 h. Sessions involved mindfulness exercises including body scans, mindful stretching and mindfulness of breath and body, among others	Salivary cortisol: at baseline and after the intervention, 4 saliva samples were collected, after waking up, before lunchtime, in the late afternoon and before sleep	For the parent sample, there was a significant and moderate intervention effect on the evening cortisol
Jensen, 2012 [131]	Age range: 20–36 y. Gender, F: 66%. P: healthy meditation novices	MBSR (16)	Control groups: - An active control group (16); - A nonmanipulated inactive group (16).	Original MBSR program. There was an active control group receiving an NMSR course and a nonmanipulated inactive group	Salivary cortisol: Samples were taken at home after a practice sampling prior to the sampling day. A total of 5 samples were taken: sample 1 upon awakening and samples 2–5 every 15 min for the subsequent hour	The MBSR group showed significantly decreased cortisol secretion and significantly lower secretion than the inactive controls (post-treatment). From pre- to post-test, cortisol secretion was significantly reduced in the MBSR group compared to the inactive controls
MacDonald, 2018 [132]	Age: 25.9 y. Gender, F: 31.25%. P: highly trained wheelchair- basketball players	MT group of highly trained wheelchair-basketball players (8)	Control group of highly trained wheelchair-basketball players (8)	An 8-week intervention was implemented to coincide with a 7-week competition period; the control group was notified that they would receive education and have access to the TM intervention and a smartphone application	Salivary cortisol: Measurements in the morning, at the same time of day, on the same day of the week. The competition and training schedule allowed participants to avoid strenuous exercise 24 h prior to saliva collection	Salivary cortisol concentrations increased and remained elevated in the control group in response to the competition period, and recovered following the conclusion of the competition period. Conversely, although salivary cortisol increased in the MT group from MT-baseline to MT-2 weeks, it returned to a concentration no different to those at MT-baseline and at MT-4 wk and remained the same for the rest of the intervention period
Nyklicek, 2013 [133]	Mean age: 46.1 y. Gender, F: 70.6%. P: healthy community-dwelling individuals	MBSR (44)	Wait-list (44)	Original MBSR program	Participants completed a negative affect scale, after which they were subjected to the three parts of the experiment in front of a computer screen in fixed order: (1) a “Baseline” resting period (35 min), (2) a “Stress” period (12 min) and (3) a “Recovery” resting period (10 min). Salivary cortisol measurements were taken (1) after the baseline resting period; (2) after the recovery resting period (22 min post-stressor onset), assessing stress-related levels as cortisol peaks with a delay; and (3) after the detachment of equipment and debriefing (32 min after stressor onset)	During the experimental phase, cortisol levels showed a significant decrease; however, no differences were found between the groups or in the AUC of cortisol
Repo, 2022 [134]	81.37% aged between 19 and 30 years. Gender, F: 72.54%. P: undergraduate students of medicine, dentistry, psychology and logopaedics	Face-to-face MT based on the Mindfulness Skills for Students course (40)	Two groups: - A web-based Student Compass program using mindfulness and ACT (22); - A control group that received mental health support as usual (40)	The face-to-face mindfulness course had a mindfulness teacher who met the students once a week for 8 weeks, whereas the online ACT course participants met the teacher face-to-face only twice, at the beginning and at the end of the intervention	Hair cortisol: hair samples were collected on separate occasions, at baseline, post-intervention and after the 4-month follow-up	The average cortisol value decreased significantly in all participants throughout the study; however, no differences between the groups evaluated were found
Rosenkranz, 2013 [135]	Age: 45.89 y (range 19–59 y). Gender, F: 79.59%. P: healthy participants (community volunteers)	MBSR (24)	HEP (25)	Original MBSR program. For the control group, an active comparison intervention, such as a supportive group atmosphere, expert instruction and engaging in activities that are believed to provide benefits, was designed (HEP)	Salivary cortisol: five saliva samples taken at home 5 times per day (upon awakening, 30 m post-awakening, before lunch, at 3 p.m. and before bed) for 3 days to provide measures of the cortisol diurnal rhythm	Despite the group difference in the change in cortisol slope following training, no differences between the groups evaluated were found. The cortisol response diminished across time for both groups to similar extents
Sousa, 2021 [136]	Age: 24.15 y. Gender, F: 50%. P: university students	BMT (20)	Active control group	In the BMT group, an audio-guided meditation focused on the body and breathing sensations. The active control group listened to audio containing educational health information for about 15 min. The interventions lasted for 30 min a day, totaling 90 min of intervention for each group	Plasma cortisol: blood samples were collected on the 1st and 3rd days of training between 8:00 and 9:00 A.M. after 45 min of rest	A significant reduction in cortisol levels post-intervention was found in the active control group
DaSilva, 2023 [137]	Mean age: 40.95 y. Gender: not reported. P: technical–administrative university workers	MBSR (15)	Wait-list (15)	Original MBSR program. In addition, there was a 4 h immersion session in a larger space with an added natural area	Hair cortisol samples were collected 2 weeks before the beginning of the study and again after the end of the study (12 weeks after the first data collection)	Participants in the MBSR group had their hair cortisol levels significantly reduced

**Abbreviations:** ACT: acceptance and commitment therapy, AUC: area under curve, BCS: breast cancer survivors, BMT: brief mindfulness training, CAR: cortisol awakening response, FFSE: freedom from smoking enhanced, HEP: health enhancement program, HRVB: heart rate variability–biofeedback, KMBSR: MBSR applied to the Korean population, LDMBI: low-dose mindfulness-based intervention, LDMBSR: low-dose mindfulness-based stress reduction, LTMs: long-term meditators, MBAT: mindfulness-based art therapy, MBCT: mindfulness-based cognitive therapy, MBCUL: mindfulness-based coping with university life, MB-EAT: mindfulness-based eating awareness training; MBIs: mindfulness based interventions; MBRT: mindfulness-based resilience training; MBSR: mindfulness-based stress reduction; MBSRbc: MBSR for breast cancer; MBX: mindfulness-based stretching and deep breathing exercise; MT: mindfulness training; MTS: mindfulness training for smokers; NMSR: nonmindfulness stress reduction; PCBMP: primary care brief mindfulness practice; P: population; PTSD: post-traumatic stress disorder; SET: supportive expressive therapy; SMC: stress management course; SMS: stress management seminar; TAC: time/attention control; TAU: treatment as usual; y: years.

**Table 3:** Summary of results from studies without significant changes in cortisol levels, with MBIs as interventions (n = 10).

Author, Year [Ref.]	Subjects: Age in Years (y) and Gender; M = Male; F = Female; Population (P)	Intervention Group (n)	Intervention in Control Group (n)	Type of Intervention	Cortisol Measurements	Results
Lynch, 2011 [138]	Mean ages: 34.30 y (intervention group) and 28.83 y (control group). Gender, F: 75%. P: university students	MBCUL (10)	Wait-list (6)	MBCUL	Salivary cortisol: 2 consecutive days (eight samples). The first sample was taken in bed upon awakening; then 15 min later, 30 min later and 45 min after awakening; and then at 9, 13, 17 and 21 h (AUC and CAR)	No significant changes were found
Bowden, 2012 [139]	Age: 34 y. Gender, F: 63.64%. P: healthy participants	Mindfulness (12)	Yoga (12) or BWV (12)	Yoga: 24.2 h (two 75 min sessions for 5 consecutive weeks; practice at home for 10 min each day). BWV: 24.2 h (two 75 min sessions for 5 consecutive weeks; practice at home for 10 min each day)	Salivary cortisol: 2 samples taken after 10 and 40 min into the assessment pre- and post-intervention between 11 a.m. and 3 p.m.	No significant changes were found
Gex-Fabry, 2012 [140]	Mean age: 47.5 y (range: 24–66 y). Gender, F: 71.42%. P: patients remitted with recurrent depression	MBCT + TAU (28)	TAU (28)	MBCT. Participants in the TAU group had unrestricted access to any type of treatment or help, or MBCT plus TAU	Salivary cortisol: 7 samples taken per day at home (upon awakening; at 15, 30, 45 and 60 min post-awakening; and at, 3 P.M. and 8 P.M.) on 6 occasions	No significant changes were found
Malarkey, 2013 [141]	Mean age: 50 y. Gender, F: 87.5%. P: university faculty and staff	LDMBI (93)	Education control (93)	LDMBI. The education control group was a lifestyle education group, which received an information-based approach enabling individuals to make health decisions based on scientific health information	Salivary cortisol: Before randomization and after the 8-week intervention. Additionally, measurements were taken on 3 days (days 2, 8 and 14 of a 2-week period) at 20 min post-rising, noon, 5 p.m. and bedtime	No significant changes were found
Fendel, 2021 [142]	Age: 31.02 y. Gender, F: 65.31%. P: resident physicians	MBSR (77)	Mindfulness description booklet (73). This group received the same course as the intervention group, except that the control group did not receive a description of practical exercises	Original MBSR program	Hair cortisol: hair samples 1 cm in length and 3 mm in diameter close to the scalp from a posterior vertex position at four on-site appointments	No significant changes were found
Graham, 2022 [143]	Median age: 33 y. Gender, F: 93.4%. P: healthy nurses	Online MBSR (31)	Relaxing music (30)	In the intervention group, the program included weekly introductions to the concept of guided meditations (available on the website or via the iPhone app). The control group was given a link to a website where they could download digital recordings of relaxing music and were instructed to quietly listen to one or more of the recordings for at least 5 min every day for 6 weeks while doing nothing else.	Salivary cortisol: at baseline and after 6 weeks	No significant changes were found
Hilcove, 2021 [144]	Mean age: 42.45 y. Gender, F: 94.85%. P: nurses and healthcare professionals	Mindfulness-based yoga (41)	The control group did not receive the yoga intervention (39)	The intervention group started with seated centering, a brief teaching about yoga, focused attention on the breath and yogic breath practice. The control group did not receive the intervention (duration of study, 6 weeks)	Salivary cortisol: Before and after the 6-week intervention. Samples were collected three times per day over two days	No significant changes were found
Oken, 2010 [145]	Age: 64.46 y. Gender, F: 80.64%. P: community-dwelling caregivers	Mindfulness meditation intervention adapted from the MBCT (10)	Two groups: - An education class based on powerful tools for caregivers, serving as an active control group (11); - A respite-only group serving as a pragmatic control (10)	The two active interventions lasted 7 weeks and consisted of one 90 min session per week along with at-home implementation of the knowledge learned	Salivary cortisol: 3 times during the assessment day, within 5 min after awakening, 30 min later before eating and at bedtime	No significant changes were found
Rosenkranz, 2016 [146]	Mean age: 49.35 y. Gender, F: 61.76. P: experienced meditators	LTMs (31). In this group, the practices reflected the progression of foundational skills taught in standard MBSR courses, and can thus be viewed in many ways as a long-term and more in-depth extension of MBSR	Meditation-naïve participants (37)	The LTM group undertook at least three years of vipassana and compassion/loving-kindness meditation, with daily practice of 30 min or more, as well as 3 or more intensive meditation retreats lasting 5 or more days	Salivary cortisol: immediately after the end of the “TSST”, as well as every 10 min for the next 40 min, for a total of 6 saliva samples	No significant changes were found
Turner, 2020 [147]	Age ≥ 17 y (88.88% between 17 and 30 y). Gender, F: 70.37%. P: students exposed to exam stress	Mindfulness Skills for Students (27)	Support as usual (27)	11 h (8 weekly sessions lasting 75–90 min)	Blood cortisol: one sample; no timing reported	No significant changes were found

**Abbreviations:** AUC: area under curve; BWV: brain wave vibration; CAR: cortisol awakening response; CBT: cognitive behavioral therapy; LDMBI: low-dose mindfulness-based intervention; LTMs: long-term meditators; MBCT: mindfulness-based cognitive therapy; MBCUL: mindfulness-based coping with university life; MBSR: mindfulness-based stress reduction; P: population; TAU: treatment as usual; TSST: the Trier Social Stress Test; y: years.

## Data Availability

The data that support the findings of this review are available from the corresponding author upon reasonable request.

## Supplementary Materials

The following supporting information can be downloaded at: https://www.mdpi.com/article/10.3390/neurolint16060115/s1, Table S1: PRISMA 2020 Main Checklist.

## References

[1] Kim D.J. (2022). Mapping the mindfulness: An literature Review of mindfulness in educational field. Open Educ. Stud..

[2] Baer R., Crane C., Miller E., Kuyken W. (2019). Doing no harm in mindfulness-based programs: Conceptual issues and empirical findings. Clin. Psychol. Rev..

[3] Goldberg S.B., Anders C., Stuart-Maver S.L., Kivlighan D.M. (2023). Meditation, mindfulness, and acceptance methods in psychotherapy: A systematic review. Psychother. Res..

[4] Bishop S.R., Lau M., Shapiro S., Carlson L., Anderson N.D., Carmody J., Segal Z.V., Abbey S., Speca M., Velting D. (2004). Mindfulness: A proposed operational definition. Clin. Psychol. Sci. Pract..

[5] Anālayo B. (2019). Adding historical depth to definitions of mindfulness. Curr. Opin. Psychol..

[6] Kabat-Zinn J. (1990). Full Catastrophe Living: Using the Wisdom of Your Mind and Body to Face Stress, Pain, and Illness.

[7] Creswell J.D. (2017). Mindfulness Interventions. Annu. Rev. Psychol..

[8] Srour R.A., Keyes D. (2024). Lifestyle Mindfulness In Clinical Practice.

[9] Segal Z.V., Williams J.M.G., Teasdale J. (2013). Mindfulness-Based Cognitive Therapy for Depression: A New Approach to Preventing Relapse.

[10] Sipe W.E., Eisendrath S.J. (2012). Mindfulness-based cognitive therapy: Theory and practice. Can. J. Psychiatry.

[11] Derlic D. (2022). From Cognitive Behavioral Therapy to Mindfulness-Based Interventions. J. Correct. Health Care.

[12] Kripalani S., Pradhan B., Gilrain K.L. (2021). The potential positive epigenetic effects of various mind-body therapies (MBTs): A narrative review. J. Complement. Integr. Med..

[13] Heckenberg R.A., Eddy P., Kent S., Wright B.J. (2018). Do workplace-based mindfulness meditation programs improve physiological indices of stress? A systematic review and meta-analysis. J. Psychosom. Res..

[14] Gu J., Strauss C., Bond R., Cavanagh K. (2015). How do mindfulness-based cognitive therapy and mindfulness-based stress reduction improve mental health and wellbeing? A systematic review and meta-analysis of mediation studies. Clin. Psychol. Rev..

[15] Alsubaie M., Abbott R., Dunn B., Dickens C., Keil T.F., Henley W., Kuyken W. (2017). Mechanisms of action in mindfulness-based cognitive therapy (MBCT) and mindfulness-based stress reduction (MBSR) in people with physical and/or psychological conditions: A systematic review. Clin. Psychol. Rev..

[16] Goldberg S.B., Tucker R.P., Greene P.A., Davidson R.J., Wampold B.E., Kearney D.J., Simpson T.L. (2018). Mindfulness-based interventions for psychiatric disorders: A systematic review and meta-analysis. Clin. Psychol. Rev..

[17] Hoge E.A., Bui E., Mete M., Dutton M.A., Baker A.W., Simon N.M. (2023). Mindfulness-Based Stress Reduction vs Escitalopram for the Treatment of Adults with Anxiety Disorders: A Randomized Clinical Trial. JAMA Psychiatry.

[18] Loucks E.B., Schuman-Olivier Z., Britton W.B., Fresco D.M., Desbordes G., Brewer J.A., Fulwiler C. (2015). Mindfulness and Cardiovascular Disease Risk: State of the Evidence, Plausible Mechanisms, and Theoretical Framework. Curr. Cardiol. Rep..

[19] Hamasaki H. (2023). The Effects of Mindfulness on Glycemic Control in People with Diabetes: An Overview of Systematic Reviews and Meta-Analyses. Medicines.

[20] Serrat M., Sanabria-Mazo J.P., Almirall M., Musté M., Feliu-Soler A., Méndez-Ulrich J.L., Sanz A., Luciano J.V. (2021). Effectiveness of a Multicomponent Treatment Based on Pain Neuroscience Education, Therapeutic Exercise, Cognitive Behavioral Therapy, and Mindfulness in Patients with Fibromyalgia (FIBROWALK Study): A Randomized Controlled Trial. Phys. Ther..

[21] Zhou B., Wang G., Hong Y., Xu S., Wang J., Yu H., Liu Y., Yu L. (2020). Mindfulness interventions for rheumatoid arthritis: A systematic review and meta-analysis. Complement. Ther. Clin. Pract..

[22] Anheyer D., Haller H., Barth J., Lauche R., Dobos G., Cramer H. (2017). Mindfulness-Based Stress Reduction for Treating Low Back Pain: A Systematic Review and Meta-analysis. Ann. Intern. Med..

[23] Ng J.Y., Anagal M., Bhowmik T. (2021). Low back pain patients’ perceived effectiveness of utilizing complementary and alternative medicine: A systematic review of qualitative studies. J. Complement. Integr. Med..

[24] Pascoe M.C., Thompson D.R., Jenkins Z.M., Ski C.F. (2017). Mindfulness mediates the physiological markers of stress: Systematic review and meta-analysis. J. Psychiatr. Res..

[25] Lao S.A., Kissane D., Meadows G. (2016). Cognitive effects of MBSR/MBCT: A systematic review of neuropsychological outcomes. Conscious. Cogn..

[26] Ni Y., Ma L., Li J. (2020). Effects of Mindfulness-Based Stress Reduction and Mindfulness-Based Cognitive Therapy in People with Diabetes: A Systematic Review and Meta-Analysis. J. Nurs. Scholarsh..

[27] Xunlin N.G., Lau Y., Klainin-Yobas P. (2020). The effectiveness of mindfulness-based interventions among cancer patients and survivors: A systematic review and meta-analysis. Support. Care Cancer.

[28] Stadnyk A., Casimiro H.J., Reis-Pina P. (2023). Mindfulness on Symptom Control and Quality of Life in Patients in Palliative Care: A Systematic Review. Am. J. Hosp. Palliat. Care.

[29] Goldsmith E.S., Koffel E., Ackland P.E., Hill J., Landsteiner A., Miller W., Stroebel B., Ullman K., Wilt T.J., Duan-Porter W. (2023). Evaluation of implementation strategies for Cognitive Behavioral Therapy (CBT), Acceptance and Commitment Therapy (ACT), and Mindfulness-Based Stress Reduction (MBSR): A systematic review. J. Gen. Intern. Med..

[30] Goldstein D.S. (2019). How does homeostasis happen? Integrative physiological, systems biological, and evolutionary perspectives. Am. J. Physiol. Regul. Integr. Comp. Physiol..

[31] Davies K.J. (2016). Adaptive homeostasis. Mol. Asp. Med..

[32] Seeley K.E., Proudfoot K.L., Edes A.N. (2022). The application of allostasis and allostatic load in animal species: A scoping review. PLoS ONE.

[33] Karatsoreos I.N., McEwen B.S. (2011). Psychobiological allostasis: Resistance, resilience and vulnerability. Trends Cogn. Sci..

[34] Chu B., Marwaha K., Sanvictores T., Ayers D. (2023). Physiology, Stress Reaction. StatPearls [Internet].

[35] Agorastos A., Chrousos G.P. (2022). The neuroendocrinology of stress: The stress-related continuum of chronic disease development. Mol. Psychiatry.

[36] Mirifar A., Keil A., Ehrlenspiel F. (2022). Neurofeedback and neural self-regulation: A new perspective based on allostasis. Rev. Neurosci..

[37] de Leeuw M., Verhoeve S.I., van der Wee N.J.A., van Hemert A.M., Vreugdenhil E., Coomans C.P. (2023). The role of the circadian system in the etiology of depression. Neurosci. Biobehav. Rev..

[38] Wyns A., Hendrix J., Lahousse A., De Bruyne E., Nijs J., Godderis L., Polli A. (2023). The Biology of Stress Intolerance in Patients with Chronic Pain-State of the Art and Future Directions. J. Clin. Med..

[39] Noushad S., Ahmed S., Ansari B., Mustafa U.H., Saleem Y., Hazrat H. (2021). Physiological biomarkers of chronic stress: A systematic review. Int. J. Health Sci..

[40] DeCaro J.A., Helfrecht C. (2022). Applying minimally invasive biomarkers of chronic stress across complex ecological contexts. Am. J. Hum. Biol..

[41] Chrousos G.P. (2009). Stress and disorders of the stress system. Nat. Rev. Endocrinol..

[42] O’Connor D.B., Thayer J.F., Vedhara K. (2021). Stress and Health: A Review of Psychobiological Processes. Annu. Rev. Psychol..

[43] Godoy L.D., Rossignoli M.T., Delfino-Pereira P., Garcia-Cairasco N., de Lima Umeoka E.H. (2018). A Comprehensive Overview on Stress Neurobiology: Basic Concepts and Clinical Implications. Front. Behav. Neurosci..

[44] Hassamal S. (2023). Chronic stress, neuroinflammation, and depression: An overview of pathophysiological mechanisms and emerging anti-inflammatories. Front. Psychiatry.

[45] Barrett T.J., Corr E.M., van Solingen C., Schlamp F., Brown E.J., Koelwyn G.J., Lee A.H., Shanley L.C., Spruill T.M., Bozal F. (2021). Chronic stress primes innate immune responses in mice and humans. Cell Rep..

[46] Tschetter K.E., Callahan L.B., Flynn S.A., Rahman S., Beresford T.P., Ronan P.J. (2022). Early life stress and susceptibility to addiction in adolescence. Int. Rev. Neurobiol..

[47] Keller-Wood M. (2015). Hypothalamic-Pituitary-Adrenal Axis-Feedback Control. Compr. Physiol..

[48] DeMorrow S. (2018). Role of the Hypothalamic-Pituitary-Adrenal Axis in Health and Disease. Int. J. Mol. Sci..

[49] Vedder H. (2007). Physiology of the Hypothalamic–Pituitary–Adrenocortical Axis. Neuroimmune Biol..

[50] Oster H., Challet E., Ott V., Arvat E., de Kloet E.R., Dijk D.-J., Lightman S., Vgontzas A., Van Cauter E. (2017). The Functional and Clinical Significance of the 24-Hour Rhythm of Circulating Glucocorticoids. Endocr. Rev..

[51] Consens F.B. (2023). Circadian Rhythm Sleep-Wake Disorders. Continuum.

[52] Herman J.P., Nawreen N., Smail M.A., Cotella E.M. (2020). Brain mechanisms of HPA axis regulation: Neurocircuitry and feedback in context. Richard Kvetnansky lecture. Stress.

[53] Rao R., Androulakis I.P. (2019). Allostatic adaptation and personalized physiological trade-offs in the circadian regulation of the HPA axis: A mathematical modeling approach. Sci. Rep..

[54] Liyanarachchi K., Ross R., Debono M. (2017). Human studies on hypothalamo-pituitary-adrenal (HPA) axis. Best Pract. Res. Clin. Endocrinol. Metab..

[55] Daimon M., Kamba A., Murakami H., Takahashi K., Otaka H., Makita K., Yanagimachi M., Terui K., Kageyama K., Nigawara T. (2016). Association Between Pituitary-Adrenal Axis Dominance over the Renin-Angiotensin-Aldosterone System and Hypertension. J. Clin. Endocrinol. Metab..

[56] Joseph J.J., Golden S.H. (2017). Cortisol dysregulation: The bidirectional link between stress, depression, and type 2 diabetes mellitus. Ann. N. Y. Acad. Sci..

[57] Allen J.O., Mezuk B., Byrd D.R., Abelson J.L., Rafferty J., Abelson J., White C., Jackson J.S. (2022). Mechanisms of cardiometabolic health outcomes and disparities: What characteristics of chronic stressors are linked to HPA-axis dysregulation?. J. Aging Health.

[58] Degroote C., von Känel R., Thomas L., Zuccarella-Hackl C., Messerli-Bürgy N., Saner H., Wiest R., Wirtz P.H. (2023). Lower diurnal HPA-axis activity in male hypertensive and coronary heart disease patients predicts future CHD risk. Front. Endocrinol..

[59] Magan D., Yadav R.K. (2019). Physiological persona differences based on stress and inflammation between meditators and healthy controls. J. Complement. Integr. Med..

[60] Gadelha M., Gatto F., Wildemberg L.E., Fleseriu M. (2023). Cushing’s syndrome. Lancet.

[61] Reincke M., Fleseriu M. (2023). Cushing Syndrome: A Review. JAMA.

[62] Ceccato F., Barbot M., Mondin A., Boscaro M., Fleseriu M., Scaroni C. (2023). Dynamic Testing for Differential Diagnosis of ACTH-Dependent Cushing Syndrome: A Systematic Review and Meta-analysis. J. Clin. Endocrinol. Metab..

[63] Husebye E.S., Pearce S.H., Krone N.P., Kämpe O. (2021). Adrenal insufficiency. Lancet.

[64] Siampanopoulou V., Tasouli E., Angelousi A. (2023). Diagnostic strategies in adrenal insufficiency. Curr. Opin. Endocrinol. Diabetes Obes..

[65] Thau L., Gandhi J., Sharma S. (2023). Physiology, Cortisol. StatPearls [Internet].

[66] Lee D.Y., Kim E., Choi M.H. (2015). Technical and clinical aspects of cortisol as a biochemical marker of chronic stress. BMB Rep..

[67] Knezevic E., Nenic K., Milanovic V., Knezevic N.N. (2023). The Role of Cortisol in Chronic Stress, Neurodegenerative Diseases, and Psychological Disorders. Cells.

[68] Vega-Beyhart A., Araujo-Castro M., Hanzu F.A., Casals G. (2023). Cortisol: Analytical and clinical determinants. Adv. Clin. Chem..

[69] Androulakis I.P. (2021). Circadian rhythms and the HPA axis: A systems view. WIREs Mech. Dis..

[70] Manosroi W., Phimphilai M., Khorana J., Atthakomol P. (2019). Diagnostic performance of basal cortisol level at 0900–1300 h in adrenal insufficiency. PLoS ONE.

[71] Levine A., Zagoory-Sharon O., Feldman R., Lewis J.G., Weller A. (2007). Measuring cortisol in human psychobiological studies. Physiol. Behav..

[72] Bioletto F., Berton A.M., Varaldo E., Cuboni D., Bona C., Parasiliti-Caprino M., Prencipe N., Ghigo E., Grottoli S., Maccario M. (2023). Development and internal validation of a predictive score for the diagnosis of central adrenal insufficiency when morning cortisol is in the grey zone. J. Endocrinol. Investig..

[73] Rosmalen J.G., Kema I.P., Wüst S., van der Ley C., Visser S.T., Snieder H., Bakker S.J. (2014). 24 h urinary free cortisol in large-scale epidemiological studies: Short-term and long-term stability and sources of variability. Psychoneuroendocrinology.

[74] Chan K.A., Lit L.C., Law E.L., Tai M.H., Yung C., Chan M.H., Lam C.W. (2004). Diminished urinary free cortisol excretion in patients with moderate and severe renal impairment. Clin. Chem..

[75] Flowers K.C., Shipman K.E. (2023). Pitfalls in the Diagnosis and Management of Hypercortisolism (Cushing Syndrome) in Humans; A Review of the Laboratory Medicine Perspective. Diagnostics.

[76] Raff H., Auchus R.J., Findling J.W., Nieman L.K. (2015). Urine free cortisol in the diagnosis of Cushing’s syndrome: Is it worth doing and, if so, how?. J. Clin. Endocrinol. Metab..

[77] Casals G., Hanzu F.A. (2020). Cortisol Measurements in Cushing’s Syndrome: Immunoassay or Mass Spectrometry?. Ann. Lab. Med..

[78] Wosu A.C., Valdimarsdóttir U., Shields A.E., Williams D.R., Williams M.A. (2013). Correlates of cortisol in human hair: Implications for epidemiologic studies on health effects of chronic stress. Ann. Epidemiol..

[79] Botschek T., Hußlein V., Peters E.M.J., Brosig B. (2023). Hair cortisol as outcome parameter for psychological and neuropsychiatric interventions—A literature review. Front. Psychiatry.

[80] Wester V.L., Reincke M., Koper J.W., Akker E.L.T.v.D., Manenschijn L., Berr C.M., Fazel J., de Rijke Y.B., A Feelders R., van Rossum E.F.C. (2017). Scalp hair cortisol for diagnosis of Cushing’s syndrome. Eur. J. Endocrinol..

[81] Wright K., van Rossum E.F.C., Zan E., Werner N., Harris A., Feelders R.A., Agrawal N. (2023). Emerging diagnostic methods and imaging modalities in Cushing’s syndrome. Front. Endocrinol..

[82] Stalder T., Steudte-Schmiedgen S., Alexander N., Klucken T., Vater A., Wichmann S., Kirschbaum C., Miller R. (2017). Stress-related and basic determinants of hair cortisol in humans: A meta-analysis. Psychoneuroendocrinology.

[83] Michaud D.S., Thomson E.M., van Oosterhout P., McNamee J.P. (2022). Hair cortisol as a viable tool for the assessment of an association between environmental noise exposure and chronic stress. J. Acoust. Soc. Am..

[84] Sharpley C.F., McFarlane J.R., Slominski A. (2011). Stress-linked cortisol concentrations in hair: What we know and what we need to know. Rev. Neurosci..

[85] Li Y., Jia W., Yan N., Hua Y., Han T., Yang J., Ma L., Ma L. (2023). Associations between chronic stress and hair cortisol in children: A systematic review and meta-analysis. J. Affect. Disord..

[86] Xie Q., Gao W., Li J., Qiao T., Jin J., Deng H., Lu Z. (2011). Correlation of cortisol in 1-cm hair segment with salivary cortisol in humans: Hair cortisol as an endogenous biomarker. Clin. Chem. Lab. Med..

[87] Short S.J., Stalder T., Marceau K., Entringer S., Moog N.K., Shirtcliff E.A., Wadhwa P.D., Buss C. (2016). Correspondence between hair cortisol concentrations and 30-day integrated daily salivary and weekly urinary cortisol measures. Psychoneuroendocrinology.

[88] Sugaya N., Izawa S., Ogawa N., Shirotsuki K., Nomura S. (2020). Association between hair cortisol and diurnal basal cortisol levels: A 30-day validation study. Psychoneuroendocrinology.

[89] Wetherell M.A., Lovell B., Smith M.A. (2015). The effects of an anticipated challenge on diurnal cortisol secretion. Stress.

[90] Bozovic D., Racic M., Ivkovic N. (2013). Salivary cortisol levels as a biological marker of stress reaction. Med. Arch..

[91] Cieszyński Ł., Jendrzejewski J., Wiśniewski P., Kłosowski P., Sworczak K. (2020). Correlation analysis of cortisol concentration in hair versus concentrations in serum, saliva, and urine. Endokrynol. Pol..

[92] Balomenaki M., Margaritopoulos D., Vassiliadi D.A., Tsagarakis S. (2022). Diagnostic workup of Cushing’s syndrome. J. Neuroendocrinol..

[93] Pruessner J., Wolf O., Hellhammer D., Buske-Kirschbaum A., von Auer K., Jobst S., Kaspers F., Kirschbaum C. (1997). Free cortisol levels after awakening: A reliable biological marker for the assessment of adrenocortical activity. Life Sci..

[94] Bowles N.P., Thosar S.S., Butler M.P., Clemons N.A., Robinson L.D., Ordaz O.H., Herzig M.X., McHill A.W., Rice S.P.M., Emens J. (2022). The circadian system modulates the cortisol awakening response in humans. Front. Neurosci..

[95] Segerstrom S.C. (2023). Physiometrics of the cortisol awakening response. Psychoneuroendocrinology.

[96] Stalder T., Kirschbaum C., Kudielka B.M., Adam E.K., Pruessner J.C., Wüst S., Dockray S., Smyth N., Evans P., Hellhammer D.H. (2016). Assessment of the cortisol awakening response: Expert consensus guidelines. Psychoneuroendocrinology.

[97] Kushner R.F., Sorensen K.W. (2013). Lifestyle medicine: The future of chronic disease management. Curr. Opin. Endocrinol. Diabetes Obes..

[98] Brown K.W., Ryan R.M., Creswell J.D. (2007). Mindfulness: Theoretical foundations and evidence for its salutary effects. Psychol. Inq..

[99] Zhang D., Lee E.K.P., Mak E.C.W., Ho C.Y., Wong S.Y.S. (2021). Mindfulness-based interventions: An overall review. Br. Med. Bull..

[100] Dimidjian S., Segal Z.V. (2015). Prospects for a clinical science of mindfulness-based intervention. Am. Psychol..

[101] Lee H.J., Stein M.B. (2023). Update on treatments for anxiety-related disorders. Curr. Opin. Psychiatry.

[102] du Plessis E.M., Just S.N. (2022). Mindfulness—It’s not what you think: Toward critical reconciliation with progressive self-development practices. Organization.

[103] Sanada K., Montero-Marin J., Díez M.A., Salas-Valero M., Pérez-Yus M.C., Morillo H., Demarzo M.M.P., García-Toro M., García-Campayo J. (2016). Effects of Mindfulness-Based Interventions on Salivary Cortisol in Healthy Adults: A Meta-Analytical Review. Front. Physiol..

[104] Aguilar-Raab C., Stoffel M., Hernández C., Rahn S., Moessner M., Steinhilber B., Ditzen B. (2021). Effects of a mindfulness-based intervention on mindfulness, stress, salivary alpha-amylase and cortisol in everyday life. Psychophysiology.

[105] Grasmann J., Almenräder F., Voracek M., Tran U.S. (2023). Only Small Effects of Mindfulness-Based Interventions on Biomarker Levels of Inflammation and Stress: A Preregistered Systematic Review and Two Three-Level Meta-Analyses. Int. J. Mol. Sci..

[106] Schuman-Olivier Z., Trombka M., Lovas D.A., Brewer J.A., Vago D.R., Gawande R., Dunne J.P.P., Lazar S.W., Loucks E.B., Fulwiler C. (2020). Mindfulness and Behavior Change. Harv. Rev. Psychiatry.

[107] Kashyap H., Mehta U.M., Reddy R.P., Bharath R.D. (2023). Role of Cognitive Control in Psychotherapy: An Integrated Review. Indian J. Psychol. Med..

[108] Hofmann S.G., Gómez A.F. (2017). Mindfulness-Based Interventions for Anxiety and Depression. Psychiatr. Clin. N. Am..

[109] Lee S.Y., Gathright E.C., Wu W.C., Salmoirago-Blotcher E. (2023). Mindfulness-Based Interventions for Patients with Cardiovascular Disease: A Focused Review for Practicing Clinicians. Curr. Cardiol. Rep..

[110] Sevilla-Llewellyn-Jones J., Santesteban-Echarri O., Pryor I., McGorry P., Alvarez-Jimenez M. (2018). Web-Based Mindfulness Interventions for Mental Health Treatment: Systematic Review and Meta-Analysis. JMIR Ment. Health.

[111] Zuo X., Tang Y., Chen Y., Zhou Z. (2023). The efficacy of mindfulness-based interventions on mental health among university students: A systematic review and meta-analysis. Front. Public Health.

[112] O’Leary K., O’Neill S., Dockray S. (2016). A systematic review of the effects of mindfulness interventions on cortisol. J. Health Psychol..

[113] Marcus M.T., Fine P.M., Moeller F.G., Khan M.M., Pitts K., Swank P.R., Liehr P. (2003). Change in Stress Levels Following Mindfulness-Based Stress Reduction in a Therapeutic Community. Addict. Disord. Their Treat..

[114] Klatt M.D., Buckworth J., Malarkey W.B. (2009). Effects of low-dose mindfulness-based stress reduction (MBSR-ld) on working adults. Health Educ. Behav..

[115] Daubenmier J., Kristeller J., Hecht F.M., Maninger N., Kuwata M., Jhaveri K. (2011). Mindfulness Intervention for Stress Eating to Reduce Cortisol and Abdominal Fat among Overweight and Obese Women: An Exploratory Randomized Controlled Study. J. Obes..

[116] Beerse M.E., Van Lith T., Stanwood G. (2020). Therapeutic psychological and biological responses to mindfulness-based art therapy. Stress Health.

[117] Matousek R.H., Pruessner J.C., Dobkin P.L. (2011). Changes in the cortisol awakening response (CAR) following participation in mindfulness-based stress reduction in women who completed treatment for breast cancer. Complement. Ther. Clin. Pract..

[118] Kim S.H., Schneider S.M., Bevans M., Kravitz L., Mermier C., Qualls C., Burge M.R. (2013). PTSD symptom reduction with mindfulness-based stretching and deep breathing exercise: Randomized controlled clinical trial of efficacy. J. Clin. Endocrinol. Metab..

[119] Bergen-Cico D., Possemato K., Pigeon W. (2014). Reductions in Cortisol Associated with Primary Care Brief Mindfulness Program for Veterans with PTSD. Med. Care.

[120] Carlson L.E., Doll R., Stephen J., Faris P., Tamagawa R., Drysdale E., Speca M. (2013). Randomized controlled trial of Mindfulness-based cancer recovery versus supportive expressive group therapy for distressed survivors of breast cancer. J. Clin. Oncol..

[121] Brinkmann A.E., Press S.A., Helmert E., Hautzinger M., Khazan I., Vagedes J. (2020). Comparing Effectiveness of HRV-Biofeedback and Mindfulness for Workplace Stress Reduction: A Randomized Controlled Trial. Appl. Psychophysiol. Biofeedback.

[122] Christopher M.S., Hunsinger M., Goerling L.R.J., Bowen S., Rogers B.S., Gross C.R., Dapolonia E., Pruessner J.C. (2018). Mindfulness-based resilience training to reduce health risk, stress reactivity, and aggression among law enforcement officers: A feasibility and preliminary efficacy trial. Psychiatry Res..

[123] Lengacher C.A., Reich R.R., Paterson C.L., Shelton M., Shivers S., Ramesar S., Pleasant M.L., Budhrani-Shani P., Groer M., Post-White J. (2019). A Large Randomized Trial: Effects of Mindfulness-Based Stress Reduction (MBSR) for Breast Cancer (BC) Survivors on Salivary Cortisol and IL-6. Biol. Res. Nurs..

[124] Errazuriz A., Schmidt K., Undurraga E.A., Medeiros S., Baudrand R., Cussen D., Henriquez M., Celhay P., Figueroa R.A. (2022). Effects of mindfulness-based stress reduction on psychological distress in health workers: A three-arm parallel randomized controlled trial. J. Psychiatr. Res..

[125] Flook L., Goldberg S.B., Pinger L., Bonus K., Davidson R.J. (2013). Mindfulness for teachers: A pilot study to assess effects on stress, burnout and teaching efficacy. Mind Brain Educ..

[126] Gardi C., Fazia T., Stringa B., Giommi F. (2022). A short Mindfulness retreat can improve biological markers of stress and inflammation. Psychoneuroendocrinology.

[127] Goldberg S.B., Manley A.R., Smith S.S., Greeson J.M., Russell E., Van Uum S., Koren G., Davis J.M. (2014). Hair cortisol as a biomarker of stress in mindfulness training for smokers. J. Altern. Complement. Med..

[128] Jedel S., Hoffman A., Merriman P., Swanson B., Voigt R., Rajan K.B., Shaikh M., Li H., Keshavarzian A. (2014). A randomized controlled trial of mindfulness-based stress reduction to prevent flare-up in patients with inactive ulcerative colitis. Digestion.

[129] Jung H.Y., Lee H., Park J. (2015). Comparison of the effects of Korean mindfulness-based stress reduction, walking, and patient education in diabetes mellitus. Nurs. Health Sci..

[130] Ho R.T.H., Lo H.H.M., Fong T.C.T., Choi C.W. (2020). Effects of a Mindfulness-based Intervention on diurnal cortisol pattern in disadvantaged families: A randomized controlled trial. Psychoneuroendocrinology.

[131] Jensen C.G., Vangkilde S., Frokjaer V., Hasselbalch S.G. (2012). Mindfulness training affects attention--or is it attentional effort?. J. Exp. Psychol. Gen..

[132] MacDonald L.A., Minahan C.L. (2018). Mindfulness training attenuates the increase in salivary cortisol concentration associated with competition in highly trained wheelchair-basketball players. J. Sports Sci..

[133] Nyklíček I., Mommersteeg P.M., Van Beugen S., Ramakers C., Van Boxtel G.J. (2013). Mindfulness-based stress reduction and physiological activity during acute stress: A randomized controlled trial. Health Psychol..

[134] Repo S., Elovainio M., Pyörälä E., Iriarte-Lüttjohann M., Tuominen T., Härkönen T., Gluschkoff K., Paunio T. (2022). Comparison of two different mindfulness interventions among health care students in Finland: A randomised controlled trial. Adv. Health Sci. Educ. Theory Pract..

[135] Rosenkranz M.A., Davidson R.J., Maccoon D.G., Sheridan J.F., Kalin N.H., Lutz A. (2013). A comparison of mindfulness-based stress reduction and an active control in modulation of neurogenic inflammation. Brain Behav. Immun..

[136] Sousa G.M., Lima-Araújo G.L., Araújo D.B., Sousa M.B.C. (2021). Brief mindfulness-based training and mindfulness trait attenuate psychological stress in university students: A randomized controlled trial. BMC Psychol..

[137] Gherardi-Donato E.C.d.S., Gimenez L.B.H., Fernandes M.N.d.F., Lacchini R., Júnior E.B.C., Díaz-Serrano K.V., Melchior M., Pérez R.G., Riquelme-Galindo J., Reisdorfer E. (2023). Mindfulness Practice Reduces Hair Cortisol, Anxiety and Perceived Stress in University Workers: Randomized Clinical Trial. Healthcare.

[138] Lynch S., Gander M.L., Kohls N., Kudielka B., Walach H. (2011). Mindfulness-based coping with university life: A non-randomized wait-list-controlled pilot evaluation. Stress Health.

[139] Bowden D., Gaudry C., An S.C., Gruzelier J. (2012). A comparative randomised controlled trial of the effects of brain wave vibration training, iyengar yoga, and mindfulness on mood, well-being, and salivary cortisol. Evid. Based Complement. Altern. Med..

[140] Gex-Fabry M., Jermann F., Kosel M., Rossier M.F., Van der Linden M., Bertschy G., Bondolfi G., Aubry J.M. (2012). Salivary cortisol profiles in patients remitted from recurrent depression: One-year follow-up of a mindfulness-based cognitive therapy trial. J. Psychiatr. Res..

[141] Malarkey W.B., Jarjoura D., Klatt M. (2013). Workplace based mindfulness practice and inflammation: A randomized trial. Brain Behav. Immun..

[142] Fendel J.C., Aeschbach V.M., Schmidt S., Göritz A.S. (2021). The impact of a tailored mindfulness-based program for resident physicians on distress and the quality of care: A randomised controlled trial. J. Intern. Med..

[143] Graham B., Jin Y., Bazeley P., Husni E., Calabrese L.H. (2022). Online, low-volume meditation does not alter immune-related biomarkers. Brain Behav. Immun. Health.

[144] Hilcove K., Marceau C., Thekdi P., Larkey L., Brewer M.A., Jones K. (2021). Holistic Nursing in Practice: Mindfulness-Based Yoga as an Intervention to Manage Stress and Burnout. J. Holist. Nurs..

[145] Oken B.S., Fonareva I., Haas M., Wahbeh H., Lane J.B., Zajdel D., Amen A. (2010). Pilot controlled trial of mindfulness meditation and education for dementia caregivers. J. Altern. Complement. Med..

[146] Rosenkranz M.A., Lutz A., Perlman D.M., Bachhuber D.R., Schuyler B.S., MacCoon D.G., Davidson R.J. (2016). Reduced stress and inflammatory responsiveness in experienced meditators compared to a matched healthy control group. Psychoneuroendocrinology.

[147] Turner L., Galante J., Vainre M., Stochl J., Dufour G., Jones P.B. (2020). Immune dysregulation among students exposed to exam stress and its mitigation by mindfulness training: Findings from an exploratory randomised trial. Sci. Rep..

[148] Rădulescu I., Drăgoi A.M., Trifu S.C., Cristea M.B. (2021). Neuroplasticity and depression: Rewiring the brain’s networks through pharmacological therapy (Review). Exp. Ther. Med..

[149] Beaty R.E., Benedek M., Kaufman S.B., Silvia P.J. (2015). Default and Executive Network Coupling Supports Creative Idea Production. Sci. Rep..

[150] Friedman N.P., Robbins T.W. (2022). The role of prefrontal cortex in cognitive control and executive function. Neuropsychopharmacol..

[151] Bremer B., Wu Q., Álvarez M.G.M., Hölzel B.K., Wilhelm M., Hell E., Tavacioglu E.E., Torske A., Koch K. (2022). Mindfulness meditation increases default mode, salience, and central executive network connectivity. Sci. Rep..

[152] Yue W.L., Ng K.K., Koh A.J., Perini F., Doshi K., Zhou J.H., Lim J. (2023). Mindfulness-based therapy improves brain functional network reconfiguration efficiency. Transl. Psychiatry.

[153] Lau W.K., Leung M.K., Chan C.C., Wong S.S., Lee T.M. (2015). Can the neural-cortisol association be moderated by experience-induced changes in awareness?. Sci. Rep..

[154] Kamali A., Milosavljevic S., Gandhi A., Lano K.R., Shobeiri P., Sherbaf F.G., Sair H.I., Riascos R.F., Hasan K.M. (2023). The Cortico-Limbo-Thalamo-Cortical Circuits: An Update to the Original Papez Circuit of the Human Limbic System. Brain Topogr..

[155] Šimić G., Tkalčić M., Vukić V., Mulc D., Španić E., Šagud M., Olucha-Bordonau F.E., Vukšić M., Hof P.R. (2021). Understanding Emotions: Origins and Roles of the Amygdala. Biomolecules.

[156] Ennis G.E., Quintin E.M., Saelzler U., Kennedy K.M., Hertzog C., Moffat S.D. (2019). Cortisol relates to regional limbic system structure in older but not younger adults. Psychoneuroendocrinology.

[157] Henze G.I., Konzok J., Kudielka B.M., Wüst S., Nichols T.E., Kreuzpointner L. (2023). Associations between cortisol stress responses and limbic volume and thickness in young adults: An exploratory study. Eur. J. Neurosci..

[158] Barry T.J., Murray L., Fearon P., Moutsiana C., Johnstone T., Halligan S.L. (2017). Amygdala volume and hypothalamic-pituitary-adrenal axis reactivity to social stress. Psychoneuroendocrinology.

[159] Norman G.R., Monteiro S.D., Sherbino J., Ilgen J.S., Schmidt H.G., Mamede S. (2017). The Causes of Errors in Clinical Reasoning: Cognitive Biases, Knowledge Deficits, and Dual Process Thinking. Acad. Med..

[160] Wynder E.L. (1994). Investigator bias and interviewer bias: The problem of reporting systematic error in epidemiology. J. Clin. Epidemiol..

[161] Adebiyi A.O. (2010). Bias: A review of current understanding. Afr. J. Med. Sci..

[162] Jager K.J., Tripepi G., Chesnaye N.C., Dekker F.W., Zoccali C., Stel V.S. (2020). Where to look for the most frequent biases?. Nephrology.

[163] Kaemmerer M., Congard A., Le Vigouroux S., Dauvier B., Andreotti E., Antoine P. (2022). Do Mindfulness-Based Interventions Have Effects Only on Negative Aspects of Psychological Functioning? A Randomized Controlled Trial. Mindfulness.

[164] Yuan W., Beaulieu-Jones B.K., Yu K.-H., Lipnick S.L., Palmer N., Loscalzo J., Cai T., Kohane I.S. (2021). Temporal bias in case-control design: Preventing reliable predictions of the future. Nat. Commun..

[165] Almeida D.M., Piazza J.R., Stawski R.S. (2009). Interindividual differences and intraindividual variability in the cortisol awakening response: An examination of age and gender. Psychol. Aging.

[166] Peeters F., Nicolson N.A., Berkhof J. (2004). Levels and variability of daily life cortisol secretion in major depression. Psychiatry Res..

[167] Fries E., Hesse J., Hellhammer J., Hellhammer D.H. (2005). A new view on hypocortisolism. Psychoneuroendocrinology.

[168] Kudielka B.M., Wust S. (2010). Human models in acute and chronic stress: Assessing determinants of individual hypothalamus-pituitary-adrenal axis activity and reactivity. Stress.

[169] Lasikiewicz N., Hendrickx H., Talbot D., Dye L. (2008). Exploration of basal diurnal salivary cortisol profiles in middle-aged adults: Associations with sleep quality and metabolic parameters. Psychoneuroendocrinology.

[170] Lederbogen F., Kühner C., Kirschbaum C., Meisinger C., Lammich J., Holle R., Krumm B., von Lengerke T., Wichmann H.-E., Deuschle M. (2010). Salivary cortisol in a middle-aged community sample: Results from 990 men and women of the KORA-F3 Augsburg study. Eur. J. Endocrinol..

[171] Thorn L., Hucklebridge F., Esgate A., Evans P., Clow A. (2004). The effect of dawn simulation on the cortisol response to awakening in healthy participants. Psychoneuroendocrinology.

[172] Thorn L., Hucklebridge F., Evans P., Clow A. (2006). Suspected non-adherence and weekend versus week day differences in the awakening cortisol response. Psychoneuroendocrinology.

[173] Persson R., Garde A.H., Hansen Å.M., Österberg K., Larsson B., Ørbæk P., Karlson B. (2008). Seasonal variation in human salivary cortisol concentration. Chronobiol. Int..

[174] Vreeburg S.A., Kruijtzer B.P., van Pelt J., van Dyck R., DeRijk R.H., Hoogendijk W.J., Smit J.H., Zitman F.G., Penninx B.W. (2009). Associations between sociodemographic, sampling and health factors and various salivary cortisol indicators in a large sample without psychopathology. Psychoneuroendocrinology.

[175] Vetter T.R., Mascha E.J. (2017). Bias, Confounding, and Interaction: Lions and Tigers, and Bears, Oh My!. Anesth. Analg..

[176] Faber J., Fonseca L.M. (2014). How sample size influences research outcomes. Dent. Press J. Orthod..

[177] Jurek A.M., Greenland S., Maldonado G. (2008). How far from non-differential does exposure or disease misclassification have to be to bias measures of association away from the null?. Int. J. Epidemiol..

[178] Andrade C. (2013). Signal-to-noise ratio, variability, and their relevance in clinical trials. J. Clin. Psychiatry.

[179] Rogerson O., Wilding S., Prudenzi A., O’Connor D.B. (2024). Effectiveness of stress management interventions to change cortisol levels: A systematic review and meta-analysis. Psychoneuroendocrinology.

[180] Lee A., Jang S., Lee S., Park H.K., Kim I.Y., Ahn R., Seok J.H., Lee K.R. (2024). Comparative analysis of salivary cortisol measurements using different assay methods in relation to serum-free cortisol measurement. Pract. Lab. Med..

