# Supplementary figures and images for: Mindfulness-Based Interventions and the Hypothalamic–Pituitary–Adrenal Axis: A Systematic Review
Source: Neurol Int. 2024 Nov 20;16(6):1552–84. doi: 10.3390/neurolint16060115 (PMC11587421; doi:10.3390/neurolint16060115)

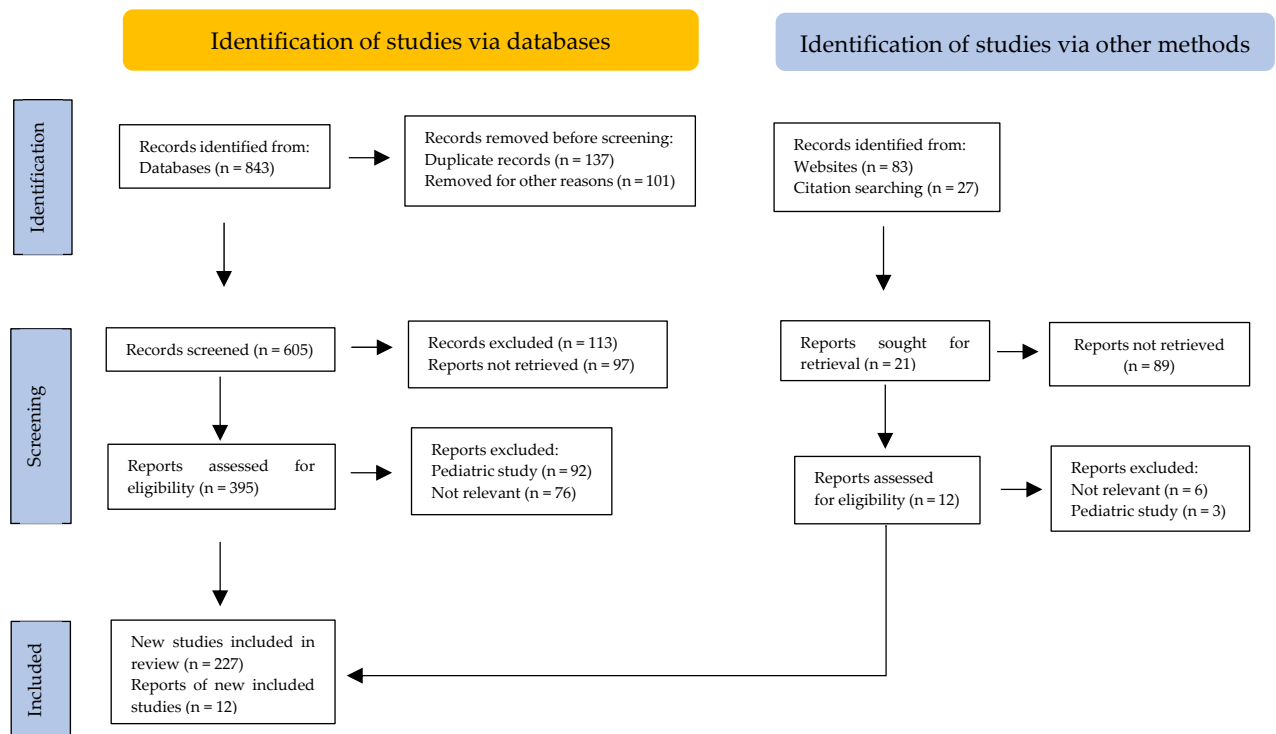

**Figure 1.** PRISMA flow diagram. Method for the selection of articles.

Supplement: Supplementary file 1 [file neurolint-16-00115-s001.zip › Figure 1. PRISMA.pdf]
